# Reconstruction of private genomes through reference-based genotype imputation

**DOI:** 10.1186/s13059-023-03105-6

**Published:** 2023-12-06

**Authors:** Matthew J. Mosca, Hyunghoon Cho

**Affiliations:** 1 Broad Institute of MIT and Harvard, Cambridge, MA USA; 2 Section of Biomedical Informatics and Data Science, Yale School of Medicine, New Haven, CT USA

**Keywords:** Genotype imputation, Imputation server, Genomic privacy, Privacy risk assessment, Reconstruction attack, Genomic data protection

## Abstract

**Background:**

Genotype imputation is an essential step in genetic studies to improve data quality and statistical power. Public imputation servers are widely used by researchers to impute their data using otherwise access-controlled reference panels of high-fidelity genomes held by these servers.

**Results:**

We report evidence against the prevailing assumption that providing access to panels only indirectly via imputation servers poses a negligible privacy risk to individuals in the panels. To this end, we present algorithmic strategies for adaptively constructing artificial input samples and interpreting their imputation results that lead to the accurate reconstruction of reference panel haplotypes. We illustrate this possibility on three reference panels of real genomes for a range of imputation tools and output settings. Moreover, we demonstrate that reconstructed haplotypes from the same individual could be linked via their genetic relatives using our Bayesian linking algorithm, which allows a substantial portion of the individual’s diploid genome to be reassembled. We also provide population genetic estimates of the proportion of a panel that could be linked when an adversary holds a varying number of genomes from the same population.

**Conclusions:**

Our results show that genomes in imputation server reference panels can be vulnerable to reconstruction, implying that additional safeguards may need to be considered. We suggest possible mitigation measures based on our findings. Our work illustrates the value of adversarial algorithms in uncovering new privacy risks to help inform the genomics community towards secure data sharing practices.

**Supplementary Information:**

The online version contains supplementary material available at 10.1186/s13059-023-03105-6.

## Background

The amount of genomic data collected and utilized for various purposes has been increasing rapidly in recent years. The largest direct-to-consumer genetic testing companies together have genotyped more than 38 million individuals [1]. In the research realm, efforts like the UK Biobank [2] and the All of Us Research Program [3] each have collected hundreds of thousands of genetic samples, for the purpose of broadly advancing science. This abundance of genomic data brings much good to society, but due to the sensitivity of genetic information and the potential for its misuse, a fundamental issue arises of a balance that must be struck between its utility and its protection. Restrictions on the use and sharing of genomic data in research, put in place to serve this protective role, have led to systems in which most genomic databases are access-controlled, only publicly shared in limited forms if at all, for example, in the form of summary-level data [4]. Various tools have emerged to facilitate this limited sharing.

A prominent example of such tools is the genotype imputation server. An imputation server is a web-based service that facilitates genotype imputation, a process by which samples genotyped on an array are algorithmically assigned genotypes at untyped (or missing) positions via comparison to a reference panel of deeply sequenced genomes. Genotype imputation is a key step in genetic association studies to improve data quality and statistical power and as a result is performed widely [5]. Existing imputation servers, such as the Michigan Imputation Server (MIS) [6] and the TOPMed Imputation Server (TIS) [7], were conceived for two primary purposes intended to provide significant value: to improve the user experience of researchers performing imputation, by simplifying technical aspects and to provide convenient public (indirect) access to large reference panels, many of which are otherwise access-controlled. It was argued that the latter aspect “eliminates the need for cumbersome data access agreements” [6]. We believe that these imputation servers genuinely do provide great value to researchers and significantly ease the process of running imputation, and the research community appears to agree; as of June 2023, more than 150 million genomes have been imputed between MIS and TIS [8, 9], and there are several imputation servers based on other access-controlled datasets, including the Sanger Imputation Service [10] and the ChinaMAP Imputation Server [11].

However, the greater convenience provided by imputation servers may come at the expense of privacy protection. Imputation fills the gaps in input data, intuitively, by copying genotypes from the most similar reference panel sequences, and therefore imputation inherently reveals some information about the underlying reference genomes. The starting point of our work was the question: *How much?*

Following other studies that have demonstrated the vulnerability of genomic data even when shared in limited forms [12–14], our objective in this investigation was to determine what portion of the reference panel (if any) can be reconstructed based on imputation server output, with the broader goal of evaluating whether there is a substantial risk to be addressed. We focused our analysis on the latest version (v4) of the minimac imputation algorithm [15] used by most imputation servers, including MIS and TIS, but also surveyed three other prominent imputation algorithms, including the PBWT algorithm [16] used by the Sanger Imputation Service.

Given the sensitive nature of the vulnerability investigated, we took great care to conduct this work responsibly and ethically. To ensure that we did not expose database participants to any additional risk, we conducted our imputation tests only with reference panels to which we already had full access. In order not to place any substantial burden on a live imputation server, we did not conduct any experiments directly on a server; rather, we simulated attacks by running the imputation algorithm packages used by existing servers, and only queried the servers to understand their input requirements and the analysis pipeline in the initial stage of the investigation. Finally, we informed representatives of existing imputation servers and other relevant stakeholders of our findings prior to publication of this work.

## Results

**Fig. 1:**
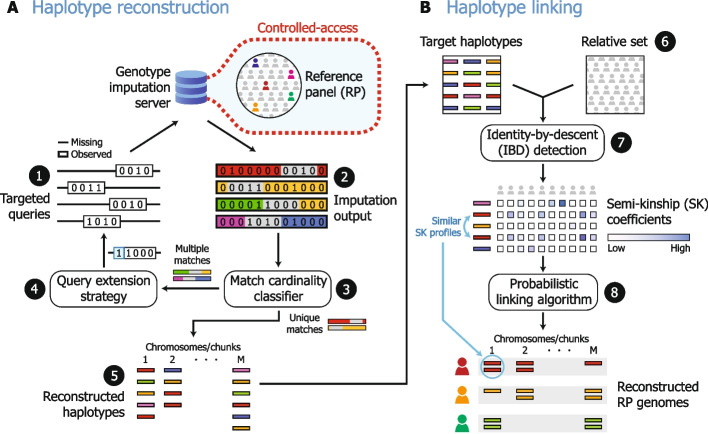
Overview of genome reconstruction attack on public imputation servers. The attack scenario we demonstrate in this work consists of two stages: haplotype reconstruction (HR; **A**) and haplotype linking (HL; **B**). In each round of HR, (1) a pool of queries targeting a short genomic region including a low-frequency variant is constructed and (2) passed to the imputation server, generating imputed data. (3) A classifier processes the output patterns to predict how many reference panel (RP) haplotypes a query matched. (4) If a query matched a small number of RP haplotypes, it is strategically extended to generate fewer matches, then passed back through imputation. (5) If a query matched a single RP haplotype, the corresponding imputed output often exactly reveals that haplotype. A set of reconstructed haplotypes (representing chromosomes or sub-chromosome chunks, depending on the configuration of the imputation server) are passed to HL. (6) HL leverages an auxiliary genomic dataset (a “relative set”) which might contain relatives of RP individuals whose data are among the reconstructed haplotype set (the “target haplotypes”). (7) HL runs an identity-by-descent (IBD) detection algorithm to get shared segments between each possible target haplotype and relative set sample pair. These segments are used to compute the semi-kinship (SK) coefficient for each pair, a measure of relatedness. (8) A probabilistic linking algorithm we developed uses these SK scores to link sets of haplotypes predicted to originate from the same individual. These correspond to RP genomes that are successfully reconstructed by the attack

We first set out to quantify the risk of data leakage by developing a potential attack against existing imputation pipelines and then evaluating its effectiveness. The attack strategy resulting from our work consists of two parts: *haplotype reconstruction* and *haplotype linking* (Fig. 1). The haplotype reconstruction portion utilizes the output from imputation to reconstruct a set of reference panel haplotypes for each chromosome or for each chromosome “chunk” (i.e., non-overlapping segments within a chromosome), depending on the pipeline configuration. (imputation is often run independently on chunks to allow for greater parallelization). The haplotype linking portion leverages any available genetic relatives to link across these genomic segments (chromosomes or chunks) to form sets of haplotypes and diplotypes predicted to belong to the same individual.

The haplotype reconstruction portion of our attack was formulated with the minimac imputation algorithm in mind and exploits the following key observation: If minimac is run on a query that exactly matches a single reference panel haplotype, the program will tend to assign to the query variants and other nearby variants the genotypes of the matching sample, with extreme dosage values. By dosage, we refer to the expected count of an allele (a continuous value between 0 and 1), where extreme values near 0 or 1 represent high confidence in a genotype prediction of 0 (reference allele) or 1 (alternative allele), respectively. Implicit in this observation is that the resulting output may contain a reconstructed portion of the matching genome and that this occurrence will be evident in the output dosages. We define an imputation “query” to be a sample sequence provided as input to the imputation pipeline, including a set of variants and the corresponding genotype assignments. Thus, a query could be a sample genotyped on an array, as in the typical use case, but could also consist of artificially constructed variants in an adversarial setting.

Our haplotype reconstruction strategy takes advantage of these insights and proceeds as follows. It samples an initial set of short queries (each including one low-frequency variant to increase the chance for a unique match), runs them through minimac, and parses the resulting output to recognize when there have been unique matches to the reference panel and reference haplotypes may have been reconstructed. Queries matching a small number of reference haplotypes (e.g., in the range of 2 to 5), also produce special dosage patterns (Additional file 1: Fig. S1; Additional file 1: Fig. S2), and when these are detected, a query-extension strategy is used to attempt to reconstruct all the matching samples.

For simplicity, we begin by analyzing the setting in which a query is imputed against the full chromosome, as opposed to separately on each chromosome chunk; later, we present analogous results for imputing chunks. In general, we observed that the imputation output of a query of only eight variants exactly matching a single 1000 Genomes Phase 3 (1KG) chromosome 20 [17] haplotype typically corresponded to the whole matching haplotype, with zero mistakes over the entire length of the chromosome (Additional file 1: Fig. S3). In other words, it was possible to perfectly reconstruct a chromosome of an individual in 1KG with only a *single* run of imputation. For more algorithmic details of our attack strategy, see the “Methods” section.

**Fig. 2:**
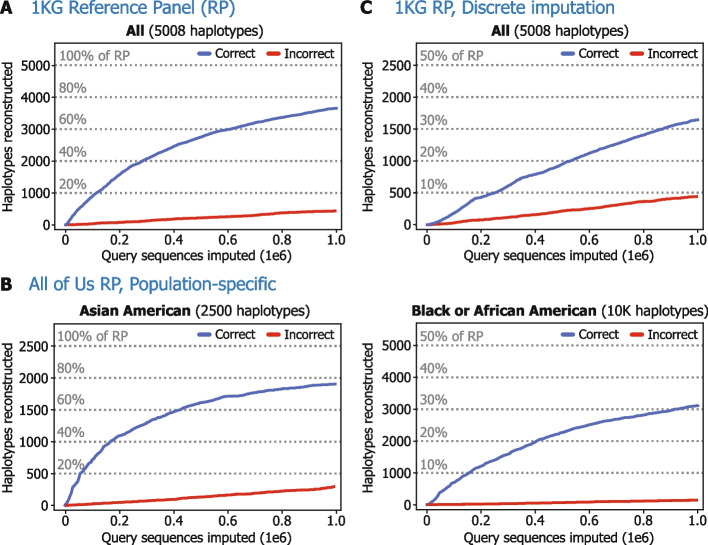
Our haplotype reconstruction strategy extracts a substantial number of chromosome-length haplotypes from imputation reference panels. We tested our haplotype reconstruction strategy, leveraging fractional dosage data output by imputation, on different reference panels (RPs), including (**A**) 1000 Genomes Phase 3 (1KG) and (**B**) two population-specific subsets of All of Us data: one including 1250 Asian American individuals and another including 5000 Black or African American individuals. **C** The “discrete imputation” version, utilizing only the discrete predictions of most likely genotype at each site, was also tested on 1KG. A reconstructed haplotype was “correct” if it had no more than 100 variants with mismatching genotypes compared to a RP haplotype and that closest haplotype had not previously been reconstructed correctly. We chose 100 as an example cutoff to allow nearly-perfect reconstructions to be considered correct, but as illustrated in Additional file 1: Fig. S4, any value in the range of 0 to 500 could likely have been chosen with little effect on the results as visualized in these plots. The count of “incorrect” haplotypes was incremented if a reconstructed haplotype had more than 100 genotype differences from the closest RP match and was sufficiently different (> 100 mismatches) from the previous incorrect haplotypes. Horizontal dotted lines represent percentages of the total number of haplotypes in a RP. In all cases, our strategy accurately reconstructed a large portion of the RP using a realistic number of queries. The results shown are for imputing chromosome 20; analogous results for imputing only the first 20-Mbp chunk of chromosome 20 are provided in Additional file 1: Fig. S9

Our haplotype reconstruction algorithm output 3656 (73%) of the 5008 haplotypes of chromosome 20 in 1KG with high accuracy (each with 100 mismatches or fewer, out of 1,047,613 variants in total) using one million artificial input samples (Fig. 2A). Ninety-six percent of all reconstructed haplotypes (including duplicates) perfectly matched a reference panel haplotype, and only 3.9% of the reconstructed haplotypes did not match any 1KG haplotype (i.e., had more than 100 mismatches) and therefore were considered incorrect. These incorrect outputs, in stark contrast to the correct ones, typically exhibited more than 1000 mismatches, while the correct outputs mostly had zero mismatches (Additional file 1: Fig. S4).

To verify that our haplotype reconstruction strategy works for reference panels different from 1KG in size and cohort composition, we also tested it on two subsets of All of Us (AoU) genomic data simulating access-controlled, ancestry-specific reference panels available on imputation servers (Methods): a panel about half the size of 1KG (1250 samples) of Asian American individuals and a panel about twice the size of 1KG (5000 samples) of Black or African American individuals [3]. For these panels, we were similarly able to reconstruct large portions of the chromosome 20 haplotypes (more than 75% and 30%, respectively) using the same number of one million queries (Fig. 2B).

Note that the apparent diminishing return in terms of the number of new haplotypes reconstructed over the course of each attack is due to the initial target variants of the queries being chosen “blindly,” that is, without knowledge of haplotypes that have already been reconstructed, which results in increasing frequency of redundant outputs. A more sophisticated query selection approach may minimize redundancy to further increase the effectiveness of the attack.

We conducted additional reconstruction experiments to study the factors that underlie performance differences in a range of reference panels. Comparing panels of different sizes sampled from the same cohort (1KG) indicated that a larger panel size tends to increase the rate of correct reconstruction and reduce error (Additional file 1: Fig. S5). We believe this is likely because when a panel is larger, more constructed queries will have a unique match relative to those with no matches; misclassification of the latter as uniquely matching queries is the primary source of error in our setting. On the other hand, holding panel size constant and varying panel ancestry composition revealed that more homogeneous ancestry-specific cohorts, together with more accurate estimates of allele frequencies in the panel, may boost reconstruction performance by improving the uniqueness of query seeds (Additional file 1: Fig. S6). We provide a more detailed discussion of these results in Additional file 1: Supplementary Note 1.

The haplotype reconstruction strategy described above hinges on interpreting the fractional (expected) dosage output by imputation, which can also be calculated from genotype probabilities if those are included in the imputation output. Although imputation could be configured (albeit at the expense of some utility) to provide only discrete genotype predictions (i.e., 0 or 1), we identified a reconstruction strategy that can circumvent this limitation (Methods). This modified strategy is based on the following insight: If a query perfectly matching a single reference haplotype is imputed, then any subsequent queries extending the original query by including additional variants from the output will tend to have identical imputation results, since the perfect match is likely preserved. This property allows an adversary to identify queries with a unique match to a reference panel haplotype. We evaluated our modified reconstruction strategy on chromosome 20 of 1KG and were again able to reconstruct a large share of the 1KG haplotypes (Fig. 2C), though at a lower reconstruction rate per input query sequence (e.g., about 425 reconstructed haplotypes for 200,000 input queries).

To test whether our findings would apply to imputation servers using alternative imputation tools in addition to minimac, we tested the discrete-genotype version of our reconstruction algorithm against IMPUTE5, Beagle5.4, and PBWT software [16, 18, 19]. The attack remained effective, with varying proportions of correct to incorrect reconstructed haplotypes, for all algorithms tested (Additional file 1: Fig. S7). While IMPUTE and Beagle share some similarity with minimac in that all three methods use hidden Markov models (HMMs) [20] as a key component, they take different approaches for utilizing the HMMs. Moreover, PBWT adopts a fundamentally different problem formulation based on set-maximal matches between pairs of sequences. Thus, our results suggest that the threat of data leakage in the imputation output is inherent to the task of imputation and not confined to specific algorithms.

In the current settings of MIS and TIS, imputation is performed separately on each chromosome chunk of 20 Mbp (i.e., the first chunk spanning base positions 1 to 20M, the second chunk spanning 20M+1 to 40M, etc.). This means that a short query, which falls within a single chunk, will result in imputation output only over that chunk. The reconstruction attack described above translates easily into this setting: Applied to the first chunk of chromosome 20, about 68% and 34% of the haplotypes in the reference panel could be reconstructed after one million queries with the fractional dosage and the discrete genotype output, respectively (Additional file 1: Fig. S8). As queries for the different chunks of a chromosome could be batched into the same input file, this setting does not necessarily increase the number of interactions with an imputation server required to retrieve a comparable amount of data, although the retrieved data are more fragmented.

The output of our haplotype reconstruction algorithm is a set of haplotypes corresponding to reference panel samples. But even if executed with perfect accuracy and all haplotypes were recovered (for all 20-Mbp chunks in the case of MIS and TIS), it would still not be known to the adversary which *group* of haplotypes corresponds to the same individual. We sought to determine whether an adversary could also infer this information. To this end, we show that the reconstructed chunks corresponding to an individual may all exhibit quantifiable relatedness to the genome of a genetic relative and thus could be linked.

**Fig. 3:**
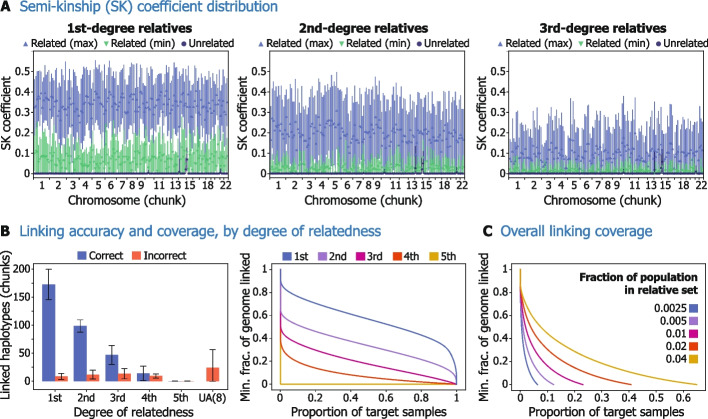
Our haplotype linking strategy leverages shared relatedness patterns across genomic regions to link reconstructed haplotypes from the same individual. We first visualize the distribution of semi-kinship (SK) coefficients across different degrees of relatedness (1st, 2nd, and 3rd), compared to unrelated individuals (**A**). SK coefficients are separately calculated for non-overlapping 20-Mbp chunks of each chromosome. Markers indicate the mean, and error bars indicate standard deviation. The distributions of the larger (max) and the smaller (min) SK values between the two target haplotypes, compared against their relative, are plotted separately. Elevated SK for related pairs distinguishes reconstructed haplotypes from the same individual, enabling them to be linked by our algorithm. **B** Left, the average number of haplotypes linked by our algorithm (out of 310 chunks in total), by degree of available relative. Error bars indicate standard deviation. “Incorrect” haplotypes refer to haplotypes assigned to the wrong individual. The rightmost bar represents unassigned (UA) sets, not assigned because the majority of haplotypes did not come from the same individual, with the number of such sets indicated in parentheses. Right, estimated proportion of individuals and their genomes which an adversary could expect to successfully link, given access to an *n*th-degree relative for those individuals. Each point (*p*, *g*) on the curve indicates that at least proportion *g* of the genome (in base pair length) could be linked for proportion *p* of the samples. These curves show smoothed cumulative distributions summarized in the bar chart (left). **C** Estimated proportion of RP individuals and the proportion of their genomes we could expect an adversary to be able to link, given access to a relative set containing a particular fraction of the population to which the RP individuals belong. Each point (*p*, *g*) on the curve indicates that at least proportion *g* of the genome (in base pair length) could be linked for proportion *p* of the samples. Our estimation leverages a population genetic model to calculate the probability of an adversary having access to relatives of different degrees, on which basis the degree-specific distributions in (B) are combined with weights

The approach we developed for this haplotype linking portion of the attack first finds identical-by-descent (IBD) segments between a set of reconstructed haplotypes, the “target set,” and an external panel of potential relatives, the “relative set.” In practice, this relative set could consist of any public or access-controlled genomic datasets to which an attacker has obtained access. The identified IBD segments are then used to compute a statistic that we define as the *semi-kinship* (SK) coefficient (Methods) for each pair of samples between the two sets. This SK coefficient indicates the expected relatedness of two individuals and is formulated specifically to compare a haploid sample with a diploid sample. The empirical distribution of the SK coefficients stratified by relatedness degree and by genomic region is summarized in Fig. 3A, which shows a consistent separation between related and unrelated pairs in all genomic regions. Note that the elevated SK values observed on chromosomes 14 and 15 for unrelated individuals are the product of likely spurious IBD segments shared by many individuals in the dataset.

These coefficients are then passed as input into a probabilistic linking algorithm we developed leveraging variational Bayesian inference and network flow optimization (Additional file 1: Fig. S9; Methods). This algorithm predicts target set chunks with a similar SK profile across the individuals in the relative set to come from the same individual. We enhance the performance of our algorithm by performing a series of preliminary steps, where SK information is combined with the results of imputing chunks against the relative set (which reveals potential IBD segments that cross chunk boundaries) to produce an initial linking solution (Methods; Additional file 1: Fig. S10 and Additional file 1: Fig. S11).

To evaluate the effectiveness of our haplotype linking algorithm, we tested it using genetic data from 2000 AoU participants, including 100 pairs of related individuals (20 pairs each of 1st- through 5th-degree relatives) and randomly sampled 900 pairs of unrelated individuals. All 44 haploid autosomal chromosomes of one individual in each pair were split into 20 Mbp chunks (310 in total) and included in the target set, for a total of 310K haplotype chunks, shuffled to obfuscate the individual of origin. The other individual in each pair was assigned to the relative set.

Using our linking algorithm, we were able to reassemble a majority of haplotype chunks from the target individuals with 1st-degree relatives in the relative set, including on average 172.8 chunks, out of the possible 310. The algorithm performed with decreased but still substantial success for individuals with more distant relatives in the relative set, for example, linking on average 98.6, 46.9, and 14.2 chunks for those with 2nd-, 3rd-, and 4th-degree relatives, respectively (Fig. 3B). An even greater portion of reference panel genomes could be reassembled if full chromosomes were returned by an imputation server, indicating that the chunking behavior provides some amount of protection against attempts to link the haplotypes: In an experiment with the same input split only into chromosome haplotypes rather than chunks, our algorithm was able to link on average 41.2, 33.7, 20.4, and 6.6 chromosomes (out of 44 haploid autosomal chromosomes in total) for target individuals with 1st-, 2nd-, 3rd-, and 4th-degree relatives, respectively (Additional file 1: Fig. S12).

Note that, in most cases beyond the 1st degree, we would only expect to obtain linked sets where each chunk is *haploid*, since most such relatives are genetically related only through one side of the family; it is therefore interesting that we were sometimes able to correctly link *both* haplotypes of a chunk: for individuals with 2nd- and 3rd-degree relatives, our algorithm on average linked about 7.8% and 3.5% of the genome (in base pair length) as diploid sequence, respectively. We believe that these results are likely explained by phasing error switching stretches of homologous chromosomes in the reference panel, an otherwise benign error (with likely minimal impact on imputation accuracy), which actually increases the power of a haplotype linking attack.

Lastly, we used a theoretical population genetic model (Additional file 1: Fig. S13; Methods) to assess the likelihood that an adversary has access to the genomic data of relatives needed for the haplotype linking portion of the attack. Using this model, we found that with access to an external database containing even 0.5% of the population to which reference panel individuals belong, at least 17% of the genome could be linked for 5% of reference panel individuals (Fig. 3C). This means that an adversary with access to any one of several existing large genomic databases could perform haplotype linking, and in theory the strategy could even leverage the database of a public third-party genetic genealogy service like GEDmatch [21], as we illustrate in Additional file 1: Fig. S14. While the theoretical model is based on a number of simplifying assumptions and should only be taken as a guideline, it indicates that this portion of the attack is feasible under realistic conditions.

## Discussion

The sum of our results reveals a novel threat vector in existing imputation services, suggesting the need for a broader community discussion on suitable mitigating measures. We would like to make explicit that our goal is not to cause alarm, but rather to share key evidence about the existence and the extent of these privacy risks to inform efforts for genomic data protection.

Our findings indicate, notably, that imputation server users who have not completed the data use agreements typically required for direct access to certain panels could still obtain access to portions of these genetic sequences using strategies similar to the ones we have demonstrated. This potentially undermines the conditions under which access-controlled genomic data were made available for use by imputation servers, which depend on the assumption that the individual-level data are sufficiently hidden from the users. Furthermore, the reference panels currently used by imputation servers include panels like the Consortium on Asthma among African-ancestry Populations in the Americas (CAAPA) panel [22] dedicated for genomes which we might believe are particularly important to protect: those of individuals who are in an underrepresented population, have a sensitive phenotype, or both. There is also the potential for the reconstruction attack to be modified to specifically target a vulnerable population, e.g., by including in queries variant alleles that are associated with rare medical conditions. The risk of reconstruction appears more significant when taking into consideration studies which have demonstrated re-identification of genomes using public databases and compelling arguments for genome-wide genetic information to be considered inherently identifiable [13, 14].

An important factor to consider when assessing the feasibility of the reconstruction attack is the computational costs involved. The runtime of haplotype reconstruction scales linearly with the number of queries imputed, and the greater part of the runtime is the time for imputation, which depends on the resources and latency of the imputation server rather than on the adversary’s resources. Based on our estimates of current MIS processing times and imputing a small batch of 128 queries at a time, processing one million queries as we did in our experiments would take approximately 10 days (Additional file 1: Fig. S15). These queries could be grouped into larger batches to run simultaneously through imputation, which would reduce the number of times an adversary would need to submit a request to the imputation server and thus further reduce the overall runtime. The upper limit on the size of each batch would be the maximum sample count allowed by the server; for MIS, this is currently 50 thousand [8]. Regarding the haplotype linking algorithm, the runtime scales approximately linearly with the number of haplotypes in the target set and with the number of samples in the relative set (Additional file 1: Fig. S16). Extrapolating based on our experiments, we would expect linking the haplotypes in a panel the size of 1KG using a relative set of 10,000 samples to take about 9.5 hours. Therefore, we believe that the reconstruction strategies we demonstrated are not overly burdensome from a computational perspective.

We recommend several measures that could be put in place to mitigate the identified risk. First, access to service could be controlled so that only approved or verified individuals can impute using reference panels that are otherwise not available to them. However, we recognize that such a measure could in implementation risk presenting a barrier to some researchers performing valuable work, so it would need to be executed with care to minimize this possible side effect. For example, one could adopt a tiered system whereby anyone with an account could still run imputation with a publicly accessible database such as 1KG. Second, rate-limitations on querying could be put in place. Although an adversary could circumvent this barrier by creating multiple accounts or colluding with other users, it could make an attack less practical given the large number of queries required to obtain the majority of a reference panel. Third, imputation servers could attempt to prevent anomalous queries by restricting the allowed input. Most simple measures (e.g., raising the minimum query size threshold) would be largely ineffective, as queries may be embedded in realistic samples to avoid detection. But a more stringent measure like requiring input to include all and only variants present in one of a set of accepted genotyping arrays has the potential to slow down the attacks we have presented at the cost of some utility due to reduced flexibility. Similarly, filtering rare variants from reference panels might slow down an attack at the cost of being able to impute those variants (Additional file 1: Fig. S17). Filtering particularly sensitive variants such as those linked to medical conditions could prevent them from being learned, also by sacrificing some utility. In general, it may be possible to recognize adversarial behavior by inspecting a user’s complete course of interaction with the server. Authentication of user identities would also aid in holding a user responsible if data reconstruction attempts are detected.

We acknowledge that these suggestions are not fully protective and that a more rigorous algorithmic solution may be desirable. For example, differential privacy (DP) [23] could be applied to reduce the degree of possible leakage, but it is not immediately clear whether a suitable trade-off between privacy and utility can be achieved using existing techniques. Furthermore, our results suggest that the exposure of *some* amount of information about the individual sequences in the reference panel is likely inherent to the task of imputation; therefore, we do not expect there will be a perfect solution that sacrifices no utility. We believe that, in the absence of such a solution, our suggestions here could help establish an acceptable balance, which recognizes both the genuine value of the imputation server and the urgency of protecting participants in a rapidly growing number of genomic data collections.

## Conclusions

In this study, we have presented evidence that genomic sequences in a reference panel used by an imputation server can be partially reconstructed by strategically querying the server and analyzing its output. Our findings suggest that the controlled-access genomic data used by existing imputation servers may not be sufficiently protected from users who do not have permission to access them directly. We have described several strategies that could be implemented by the servers to enhance privacy without overly compromising utility. We believe that our results should serve as an example of how widespread and seemingly safe data sharing practices can harbor a vulnerability and why we need efforts to assess existing and future data sharing mechanisms from an adversarial perspective. Policies that protect genomic data can only be effective to the extent that our beliefs about where risk exists are accurate.

## Methods

### Seed-sieve-extend (SSE) strategy for haplotype reconstruction

#### Relevant details about minimac

The haplotype reconstruction portion of our attack was designed with the minimac imputation algorithm used by the Michigan Imputation Server (MIS) in mind [6]. Like other imputation algorithms, minimac takes as input a set of query sequences (typically samples genotyped on an array) and uses a reference panel of sequenced samples to predict the most likely genotypes at positions where the input is untyped, outputting for each position typed in the reference panel and for each input sample haplotype a genotype prediction and a corresponding dosage value. Assuming biallelic variants, a genotype value of 0 corresponds to the reference allele (REF) and a value of 1 corresponds to the alternative allele (ALT). The output dosage value indicates, roughly, confidence in the genotype prediction. A dosage close to 0 indicates the REF allele is likely and a dosage close to 1 indicates the ALT allele is likely. Minimac has been updated over the years for the sake of space and computational efficiency, and the current state-of-the-art is minimac4 (which is the specific version of the package we use throughout), but the high-level algorithm remains the same as the original.

Minimac models the imputation problem with a hidden Markov model (HMM) such that a hidden state $S_i$ at SNP base position *i* takes on a value in $\{1, \dots , n\}$, where *n* is the number of haplotypes in the reference panel, and an observation $X_i$ represents the observed genotype and takes on a value in $\{0, 1\}$, viewed as a noisy copy of the genotype of the reference haplotype $S_i$ at the same position. Given a set of observed genotypes at a subset of positions, minimac uses the standard forward-backward algorithm to get a distribution expressing the most likely hidden states (reference haplotypes) at each untyped position. This distribution is translated into a genotype distribution based on the genotypes of the reference haplotypes, and the expected value associated with this genotype distribution corresponds to the dosage value output by the program. The discrete genotype prediction is the most likely genotype according to the distribution.

Intuitively, imputation models each query haplotype as a “mosaic” of the haplotypes in the reference panel, as if the query sequence is composed of pieces of the reference haplotypes, so that those pieces can be used to fill in the gaps in the query. This conceptualization is justified by the fact that even distantly related individuals share short stretches of DNA, known as identity-by-descent (IBD) segments. Considered this way, minimac can be thought of as finding the closest matching reference haplotype at each point along the genome. In actuality, minimac computes a probability distribution over all reference haplotypes at each position, but a haplotype that matches the query closely over a stretch of positions will be weighted heavily in the distribution at those positions.

#### Key insights enabling the strategy

The reconstruction strategy we devised hinges on a consequence of the points made just above: A query sequence that exactly matches only one single reference panel haplotype will result in genotype and dosage output that reveal information about the matching haplotype. Specifically, at positions where the query matches the haplotype and at other nearby positions, the output genotypes will tend to be those of the matching reference haplotype, and the output dosages will tend to be very close to 0 or 1 (extreme dosages), indicating “high confidence” in the genotype output. This occurs because, as noted above, a haplotype that closely matches the query at a set of positions will be heavily weighted in the distribution at those positions: in HMM terminology, no other reference haplotype is as likely to have emitted the observed genotypes in the query than the one haplotype with exactly those genotypes.

The insight described above means that when a query exactly matches a single reference panel haplotype, we may be able to recognize from the output dosages that this has occurred (a pattern of uniformly extreme dosage values is atypical) and infer that the corresponding output genotypes are likely to match a particular reference panel haplotype. In other words, if we can generate queries that exactly match the reference haplotypes, we may get reconstructed haplotypes in the imputation output and can recognize the likely success of this reconstruction. This observation provides the basis for our haplotype reconstruction strategy.

#### Overview of the approach

The high-level structure of our seed-sieve-extend (SSE) strategy for haplotype reconstruction is as follows: first, it generates a large number of short queries (“seeds”) and runs them together through minimac. Then, it utilizes a classifier operating on the output dosages to predict how many reference haplotypes the query matches. If a query is predicted to match one or a small number of reference haplotypes (say, 2 to 5, which also produces distinct dosage patterns), it proceeds with these and discards the rest (“sieving”). If the dosage pattern indicates that a uniquely matching reference chromosome or chunk has been retrieved in its entirety (imputation operates at the chromosome or subchromosome chunk level, rather than over the whole genome), the strategy has achieved its objective for that query. Alternatively, if a query matches some small number of reference haplotypes, the original query is strategically extended to attempt to retrieve each of the matching haplotypes (“extension”). This whole process is repeated with different queries and on different chromosomes/chunks to retrieve additional reference panel haplotypes. Below, we detail the attack scenario and each step of the attack, but note that values and data used in experiments, and other experiment-specific details, are discussed later in the “Haplotype reconstruction experiments” section.

#### Attack scenario

Our haplotype reconstruction strategy was devised based on the following scenario: An imputation server holds a hidden reference panel (meaning a user cannot directly access the genotype data) and an adversary’s objective is to retrieve as much of the genomic data in that reference panel as possible through interaction only via the imputation server. The adversary’s attack strategy may submit to the server as input an unlimited number of queries, where a query is a properly formatted set of variants and genotype assignments at those variants. The server’s output returned to the adversary, given an input query sequence, is the result of a minimac run with that query as input. Initially, we assume that the imputation output contains both imputed genotypes and corresponding dosages, at exactly the positions where reference panel samples are typed. Later, we also discuss an alternate strategy which assumes only discretely imputed genotypes, and not continuous dosages, are output.

#### Step 1: Seed

The first step of the strategy is to generate a large number of query sequences to be input into minimac, the goal being that some of them perfectly match one or a small number of reference panel haplotypes. These are the “seeds” around which we hope to reconstruct the reference haplotypes. In order to accomplish this goal, short queries (of, say, 8 variants) are used because a given conformation of genotypes over a large number of variants would be very unlikely to match any reference haplotype due to the exponential number of possible conformations. Specifically, the strategy repeatedly chooses a small set of variants, enumerates all possible genotype conformations on that set, and submits all of these conformations as queries. In this way, there is a guarantee that some queries will match the reference haplotypes.

Variant sets are chosen strategically in an attempt to increase the probability that some conformations match one or a small number of haplotypes. A variant with a low minor allele frequency (MAF) is chosen per set and fixed with the ALT allele, and the rest of the set is populated with variants with high MAF. The idea here is that because only a small number of haplotypes will have the ALT allele at the low-MAF SNP, it is more likely that some queries will match a small number of haplotypes. For example, if 8 haplotypes in the reference panel have the ALT allele at variant X, it is certain that at least one of the queries will exactly match between 1 and 8 haplotypes, since we are generating all possible conformations over the other set variant genotypes. High-MAF variants are chosen for the rest of the set to make it more likely that haplotypes with the ALT allele in the low-MAF variant will have different genotypes in the other set variants, resulting in fewer matches for different queries. High-MAF variants are chosen not to be too close together. Due to linkage disequilibrium, variants very near in centimorgan (cM) distance are more likely to have the same genotypes across haplotypes. However, the set variants are also chosen to be not too far apart, as we observed that variant sets with variants nearer to each other produced cleaner dosage patterns when a query sequence on those sets matched one or a small number of reference haplotypes.

Our implementation, to create each set, generates a random position within the bounds of the chromosome and attempts to build a variant set centered roughly around that position, moving on to another random position if it fails. It takes arguments specifying query length (number of set variants), allele frequency cutoffs for what counts as a low-MAF or high-MAF variant, and the minimum and maximum genetic distance spacing allowed between variants during set construction. To enable the variant spacing strategy, genetic distance values (in cM) were obtained for all variants using hg38 genetic maps provided by the authors of SHAPEIT4 and using a code adapted from theirs to interpolate genetic distances at positions not included in the maps [24]. For the variant MAF values, we used population-specific gnomAD allele frequency data [25] combined by weighted average to fit the composition of the reference panel. The specific weightings for the different reference panels used in the experiments are noted in the “Haplotype reconstruction experiments” section.

#### Step 2: Sieve

The second step of the strategy, once the query sequences have been run through minimac, is to utilize the imputation dosage output to predict, for each query, how many reference panel haplotypes it matched. As discussed above, when a query matches a small number of reference haplotypes, this fact is likely to be recognizable by specific patterns in the dosages (Additional file 1: Fig. S1; Additional file 1: Fig. S2). A random forest classifier, trained on the dosage output resulting from queries with known numbers of matches, is used to recognize these patterns and make predictions. We configured and trained the classifier to classify dosage data from the full length of the chromosome and assign a match count prediction of 1 through 5 or Other, with Other covering queries with more than five matches and also those with none.

When the classifier predicts no matches or a large number of matches (6 or more) for a query, the corresponding output is discarded. This is because the imputation outputs resulting from these queries represent a mixture of many samples, and extracting a single reference panel haplotype from these outputs is difficult. What we have left after this “sieving” is only the imputation output corresponding to queries predicted to have matched a small number of reference haplotypes (1 through 5). For queries the classifier predicted to have matched a single haplotype, we infer that a reference panel haplotype has been reconstructed, and the program saves off the corresponding output genotype data as such. The rest of the remaining queries move on to the extension step.

We used the scikit-learn RandomForestClassifier module [26] to implement the classifier. The classifier was designed to operate on bucketed dosage data, so both for training and for use in the attack, output dosage data were first processed into counts of dosages falling into each of 21 non-overlapping dosage range buckets. These buckets consisted of the following: (1) buckets of size $\alpha =0.1$ at each end (extending from 0 and from 1), (2) buckets of size $\beta =0.02$ centered at the expected values of the dosage “stripes” anticipated in the patterns for 1 to 5 matches (see the “Step 3: Extend” section for elaboration), and (3) additional buckets that span the remaining gaps in the dosage domain of 0 to 1. We used 75 decision trees in the classifier. Training data included 100 sets of dosage data from 1KG for each of the six classes (1 through 5, Other). Although our classifier serves only as an example approach to separate out the most promising queries from the rest, we proceeded with it in the rest of our experiments, since it led to successful reconstruction results across various settings, as shown in our results. An evaluation of our classifier’s performance on test data is provided in Additional file 1: Fig. S18.

#### Step 3: Extend

For queries classified as matching a small number of reference haplotypes, an extension strategy is used to attempt to retrieve all the matching haplotypes. As shown in Additional file 1: Fig. S1, the number of haplotypes a query matches corresponds to the number of “stripes” evident in the pattern of dosage values in its output. For example, a query constructed strategically (as discussed in the “Step 1: Seed” section) that matches two reference panel haplotypes will display extreme dosages and a stripe of dosages close to 0.5. This happens because the two matches are weighted more heavily along those positions, and so the output dosages are close to 0 where both have the REF allele, close to 1 where they both have the ALT allele, and close to 0.5 where one has the REF allele and the other the ALT allele. The explanation works analogously for greater numbers of matches: the output of a query matching *n* haplotypes is characterized by concentrations of dosages (“stripes”) at multiples of 1/*n*, corresponding to the proportions of REF/ALT alleles among the matches at different positions. These are the stripes that the classifier in the previous step is effectively trained to recognize.

It follows from this understanding, for the two-matches example, that if we add to the original query a variant with an output dosage near 0.5 and assign it the REF allele in a new query, the new query will match only one of the two haplotypes matching the original query. If we assign it the ALT allele in another new query, we will only match the other haplotype. In this way, we may be able to reconstruct both matching haplotypes, and this process can be generalized to a wider range of match counts.

Specifically, our program, given a query matching *n* haplotypes, chooses a nearby variant whose allele frequency falls within a specified distance from 1/*n* (in our experiments 0.025, but this distance could be optimized to be as small as possible while still reliably capturing nearby variants). We then create two new extended queries using both alleles of this variant; that is, the added variant is set to the ALT allele in one query and it is set to the REF allele in the other. The former is expected to match only one reference haplotype, as the dosage of the variant indicated only one ALT allele among the matches. The latter new query is expected to match $n - 1$ haplotypes and is further extended by choosing another variant the same way as before (now using $n - 1$ instead of *n* to determine the dosage range from which to draw the variant).

#### Alternative strategy for discrete genotype predictions

This alternative strategy assumes that imputation only outputs discrete genotypes and not continuous dosages. It hinges on the following insight: let us say we have a query that perfectly matches a single reference haplotype. If we impute on this query, form a new query by appending to the original query a nearby variant and genotype assignment from the imputation output, and impute with this new query, the output genotype predictions will tend to be the same as for the original query. This occurs because, as discussed earlier, the imputation output genotypes resulting from a query matching a single reference haplotype will tend to be those of the matching haplotype, at least at the query variants and others nearby. As a result, the variant and genotype appended to form the new query will usually also correspond to the matching haplotype, and the new query will only be a longer exact match to that haplotype. An important corollary to the above insight is that this will not necessarily happen for an initial query which does not uniquely match a reference haplotype, since the logic described will not apply.

The sum of all this points to a reconstruction strategy similar to the original that does not require continuous dosage output: First, query sequences are constructed as before and run through imputation. Then, each query sequence is extended with a variant and genotype assignment from its own imputation output, and these new query sequences are run through imputation. In our implementation, we use for extension the variant with the highest MAF below some cutoff (defaulted to 0.6) within the range stretching some base pair distance to either side of the low-MAF variant, by default 50,000 base pairs. For each extended query, the resulting output is compared to the original query’s output, and queries for which the outputs differ are filtered out. Each remaining query proceeds to the next round, in which the original version of the query is extended using a different nearby variant, and filtering is repeated in the same way. In our implementation, we choose in each round the in-range variant with the highest MAF below the cutoff that has not already been chosen. This process is repeated for *k* rounds, specified as input to the attack, or until no queries remain. Any queries remaining after the specified number of rounds are predicted to match a single reference panel haplotype, and its output can be saved as such. In the language used to describe the “standard” method for haplotype reconstruction presented first, this “discrete genotype” method can be described in general as seeding and then iteratively extending and sieving, with a sieving mechanism that does not rely on dosage values.

The chosen number of rounds of extension *k* affects the number of incorrect reconstructions that the attack makes. A greater number of rounds will catch and filter more of these errors, at the cost of increased running time, so the choice of *k* is a trade-off between priorities. We would not expect the same *k* to result in the same amount of error across the imputation tools, and in our experiments we found that different tools required different settings in order to achieve a similar quality of filtering (see the “Imputation tools” below for specific settings). We generally observed that, once sufficiently high, the value of *k* did not have much effect on performance; in practice, an adversary could further optimize it based on the rate of reconstruction as it progresses.

Although we have not explored this idea in our work, imputing a subset of variants in a sample then checking whether the output is consistent with the input could be a useful approach for distinguishing correctly reconstructed haplotypes from incorrect ones even in the fractional dosage output setting.

#### Rounds of interaction and input size limits

It is worth commenting on the number of times that the imputation software must be run in the execution of haplotype reconstruction. This aspect is relevant for an accurate understanding of the risk posed to imputation servers, since the number of times imputation is run is, in practice, the number of times an adversary must upload data to the server. We submit the query conformations on a single variant set (of 8) as a discrete unit for a run of imputation, and each following extension step requires an additional round of interaction. Thus, the overall number of rounds is at least as large as the number of rounds of extensions plus one (in our experiments at most 5 for standard reconstruction, 15 or 30 depending on the imputation tool for the discrete-genotype version). Since each set of target variants is processed independently of other target sets, they can be explored by the attacker in parallel. This implies that the total number of rounds depends on the limit imposed by the server on the maximum size of the input; the larger the limit, the more target variant sets can be included in each input to reduce the total interaction rounds. As a reference point, the maximum input sample size of MIS is currently 50,000 [8]. Optimization in terms of query sequences required per reconstructed haplotypes, via adjustment of parameters (discussed briefly below), could further reduce the number of rounds of interaction required, but this optimization was not a priority of our investigation. For minimac, we note that including different sets of target variants in a single input file has a potential impact on the behavior of the imputation by enforcing the HMM computation on the missing sites of each query (corresponding to *other* target variant sets in the same file); however, the expected difference in probabilities is small, and thus we do not view this as a major limitation of the attack.

### Haplotype reconstruction experiments

#### Datasets

For experiments carried out in 1000 Genomes Phase 3 (1KG) [17], we used phased data downloaded from the International Genome Sample Resource (IGSR). For full chromosome experiments, the full 1KG chromosome 20 VCF data were used as a reference panel. For chunk experiments, the VCF was first subset variant-wise to the bounds of the chunk (using the same 20-Mbp chunks used by MIS and TIS), and this subset file was used as the reference panel.

All of Us (AoU) [3] Controlled Tier Dataset v5 was used for experiments with AoU data. The Asian American and Black or African American panels of the AoU were populated based on the value of the “RACE” column of the database only, including participants with values “Asian” and “African,” respectively, in the following way: for the Asian American panel, we started with all 3064 Asian American AoU participants with whole genome sequencing data; used Hail [27] to filter to only chromosome 20, filter to only biallelic variants, and filter out variants with greater than 10% missingness; phased the data using Eagle2 [28]; and finally, randomly sampled a subset of the desired 1250 samples. For the Black or African American panel, we used an identical process except that the data were downsampled to 5000 participants prior to phasing, because 5000 is considered sufficiently large for accurate phasing [28].

For the variant MAF values used in reconstruction, we used population-specific gnomAD allele frequency data [25] combined by weighted average to fit the demographics of each of these reference panels. For 1KG, these (relative) weightings were 99/2015 for the Finnish allele frequency, 404/2015 for Non-Finnish European, 504/2015 for East Asian, 347/2015 for American Admixed/Latino, and 661/2015 for African/African American, where the numerators are the number of individuals in 1KG that fall into each group. 1KG also contains 489 South Asian samples, but the gnomAD v2 file used included this population in the “other” category due to the small number of South Asian samples, so we opted not to directly account for this population. For the Asian American AoU panel, the weightings were 1/2 for the East Asian allele frequency and 1/2 for South Asian (gnomAD v3.1 used here does have a separate South Asian allele frequency). For the African American AoU panel, we simply used the gnomAD v3.1 African/African American allele frequency.

#### Imputation tools

Below are the details of our use of minimac4 [6] and the other three imputation tools that we tested: Beagle5.4 [19], IMPUTE5 [18], and PBWT [16].

##### minimac4

Version 1.0.3 (also known as v4.0.3) was used. It was run with the –minRatio parameter set to 0.000001, –chunkLengthMb set to 140 (longer than chromosome 20), and the –ignoreDuplicates flag. The package accepted our 8-variant queries in their haploid form, merely inserted into VCF format. For the discrete genotype reconstruction tests, the number of extension rounds was set to 15. Updates to minimac4 have been released since we first began our experiments (currently v4.1.2), but these updates do not change our conclusions. We verified by a small test that our code is easily adapted to reconstruct successfully using the newest version by simply changing command-line syntax for calling the package, reference panel file format conversion, and indexing of the query file. This software can be found at https://github.com/statgen/Minimac4.

##### Beagle5.4

Version 22Jul22.46e was used. The reference panel was first converted to the bref3 format before being passed to the package. The window parameter was set to 110. The package accepted our queries in their haploid form in gzipped VCF format. The number of extension rounds in the reconstruction test was set at 15. This software can be found at http://faculty.washington.edu/browning/beagle/beagle.html.

##### IMPUTE5

Version 1.1.5 was used. According to its usage instructions, the reference panel was first converted to the imp5 format before being passed to the package. It was run with the –pbwt-depth parameter set to 32, –pbwt-cm set to 0.001, and the –neigh-select flag. To comply with input requirements, queries were converted to diploid form when inserted into the VCF format by simply duplicating each allele. The reconstruction test was set to use 30 rounds of reconstruction, as it seemed a greater number was needed for satisfactory performance with this tool. This software can be found at https://jmarchini.org/software/#impute-5.

##### PBWT

Version 3.0 was used. The reference panel was first converted to the pbwt format before being passed to the package. To conform to input requirements, queries were converted into diploid form when inserted into VCF format by simply duplicating each allele. The first and last variants of the chromosome were also added to query VCF files, set to the reference allele, so that matches extending the whole length of the chromosome could occur, resulting in imputed genotypes over the entire length. The extension strategy used in the reconstruction test was modified slightly for this package because its algorithm is the most distinct from the others (not using an HMM), and as a result it required a little more targeted treatment: Each round, queries were extended with multiple variants chosen from different gaps in the query, rather than with just one variant. The test was set to use 30 rounds of reconstruction, as it seemed that a greater number were needed for satisfactory performance. This software can be found at https://github.com/richarddurbin/pbwt.

#### Attack settings

For all haplotype reconstruction experiments, we used query variant sets of 8 variants (1 low-MAF variant, 7 high-MAF), low-MAF cutoff of 0.005 (variant considered low-MAF if it had MAF below this value), high-MAF cutoff of 0.2 (MAF above this value required), minimum genetic distance variant spacing of 0.001 cM, and maximum spacing of 0.0035 cM. While this parameter setting was effective for all our experiments, we did not prioritize optimization, and hence further exploration may lead to better parameter choices with a higher reconstruction performance.

Discarding all rare variants in the reference panel (e.g., below an MAF cutoff of 0.01 or 0.005) to reduce the effectiveness of the low-MAF variant in the query slows reconstruction, but only to a limited extent, as shown in Additional file 1: Fig. S17. For example, we see in this figure that around 20% of the reference panel is reconstructed for both the 0.005 and 0.01 MAF thresholds, in contrast to 30% without filtering using the same number of queries. For all experiments using the standard fractional-dosage version of reconstruction, we only considered queries constructed in the middle 80% of the chromosome (in base position), as we found that this was a simple measure that would reduce error due to boundary effects (see Additional file 1: Supplementary Note 1 for more details).

#### Accommodating MIS/TIS query requirements in practice

Although we ran our primary experiments directly on imputation packages, rather than against real imputation servers (for ethical reasons, as we discuss in the main text), in the early stages of testing, we checked whether our queries would be accepted by MIS, imputing against the 1KG reference panel. We found that two slight adjustments, compared to the code used in our experiments, were required for our queries to pass MIS quality control (QC). First, only single-nucleotide polymorphisms (SNPs) were accepted by MIS, with any indels filtered out. This is easily addressed by limiting to only SNPs the variants that the attack uses to form queries. Second, QC filtered out monomorphic sites (where all samples have the same allele). This requirement could be addressed to ensure no query variants are filtered out for this reason by including in any attack input file a dummy query which is the inverse (allele at every site flipped) of one of the real queries. In summary, we found that none of the input requirements used in practice by MIS precluded reconstruction in the way we have described, and we were able to successfully impute on the server from a sample of our constructed queries as of July 28, 2023. TIS is based on the same software as MIS and has the same QC requirements, so the same statement applies, though we did not run any queries on TIS to avoid reconstructing from any reference panel to which we did not already have access.

### Probabilistic inference algorithm for haplotype linking

The output of our reconstruction attack, performed on an imputation server such as MIS or TIS that utilizes chunking, is a collection of decoupled haplotype chunks of unknown origin. However, since the recent genetic lineage of each individual is likely to be reflected across chunks, having access to the genetic sequence of a relative of an individual can enable linkage of reconstructed haplotypes to reveal a portion of the individual’s genome. Here, we describe the probabilistic algorithm we developed to demonstrate accurate linkage of haplotypes from the same individual.

#### Capturing haplotype-diplotype relatedness via semi-kinship (SK) coefficients

To link reconstructed haplotypes, we need a way to quantify the genetic relatedness of each compared to diploid samples in an external set of potential relative genomes (the “relative set,” versus the “target set” of reconstructed haplotypes). A common measure of the genetic relatedness of two samples is the *kinship coefficient*, $\phi$ [29]. The kinship coefficient of two samples *i* and *j* is generally defined as the probability that a random allele chosen from *i* and a random allele chosen from *j* at the same locus are identical by descent (IBD). Formally, it can be defined as $\phi = k_1/4 + k_2/2$, where $k_1$ is the probability that the samples share one IBD allele at a randomly chosen locus and $k_2$ is the probability that the samples share two IBD alleles. However, this definition assumes that the two samples being compared are diploid. To quantify the relatedness of a haploid sample to a diploid one, we formulated the *semi-kinship* (SK) coefficient, $\phi _{1/2}$, which we define as $\phi _{1/2} = k_1/2$. The SK coefficient extends the notion of kinship coefficient to the haploid setting; it is the probability that an allele from a haploid sample and a randomly chosen allele from a diploid sample at the same locus are IBD.

Obtaining a SK value for each (chunk-length) pair of target set haplotype and relative set diplotype required computing $k_1$ for the pair, and to this end, we ran both sets together through GERMLINE2 [30], which computes IBD segments for every pair of haplotypes. The $k_1$ value was calculated by dividing the total genetic length (in centimorgans) shared IBD with either haplotype in the relative set sample by the total centimorgan length of the chunk.

#### Formal definition of the haplotype linking problem

In formally presenting our linking algorithm here and in the following, we consider the problem with respect to linking chromosome-length haplotypes, for simplicity. It is straightforward to modify the algorithm to link chunks rather than full chromosomes, as we did for our main linking experiment.

Let *N* be the number of haplotypes reconstructed in the target set, *T* be the number of individuals in the target set, and *M* be the number of diplotypes in the relative set. We use $S\in[0,1]^{N\times M}$ to denote the observed SK values between every haplotype-diplotype pair between the target set and the relative set. The SK values are based on true (unobserved) genetic relationships between each pair of individuals $j \in [T]$ and $\ell \in [M]$, which we represent using the variable $r_{j,\ell } \in \mathcal {A} := \{\emptyset , 1, 2, 3+\}$. Note that $[n]:= \{1,\dots ,n\}$ for an integer *n*. $\emptyset$ represents unrelated individuals, while other values of $r_{j,\ell }$ represent the *degree* of relatedness, with $3+$ corresponding to the 3rd degree or higher relatives (up to the 5th degree in our experiments to distinguish from unrelated). Let $R\in \mathcal {A}^{T \times M}$ be the matrix whose elements correspond to $r_{j,\ell }$’s.

The goal of the haplotype linking problem is to find a mapping $g:[N] \mapsto [T]\cup \{\emptyset \}$, such that for each $j\in [T]$, the set $g^{-1}(j) := \{ i: g(i) = j \}$ corresponds to a set of target haplotypes that belong to the same individual *j*. We consider $g^{-1}(\emptyset )$ to be haplotypes that could not be linked with sufficient confidence. Also, we require that $g(\cdot )$ not assign more than two haplotypes of the same chromosome to the same target individual.

#### Probabilistic generative model

To infer the mapping $g(\cdot )$ based on the observed SK values *S*, where *g* in turn reveals which haplotypes are likely to originate from the same individual, we first formulate a probabilistic model that induces a distribution over *S* given a mapping *g*, then obtain a maximum likelihood estimate of *g* conditioned on *S*. We define the probabilistic model below, illustrated in Additional file 1: Fig. S9A.

We first let $\theta _{i,j} \in \{0,1\}$ for $i\in [N]$ and $j\in [T+1]$ be the parameters defining $g(\cdot )$. We will use $g_\Theta (\cdot )$ to clarify its dependence on $\{\theta _{i,j}\}$, where $g_\Theta (i)=j$ if and only if $\theta _{i,j}=1$ for $j\in [T]$, and $g_\Theta (i)=\emptyset$ if and only if $\theta _{i,T+1}=1$. The probabilistic model is then fully specified by the variables $\Theta$, *R*, and *S*, where only *S* is observed. The generative process follows a two-step procedure: we first sample the latent variables $r_{j,\ell }$ for each $j\in [T]$ and $\ell \in [M]$ from a prior distribution $p(r_{j,\ell })$, which we allow to be controlled by an experimental variable. We then sample each $s_{i,j}$ from $p(s_{i,j}| r_{g_\Theta (i),j})$ when $g_\Theta (i)\ne \emptyset$, which represents the conditional distribution over the SK value for a given pair of individuals $(g_\Theta (i),j)$ of a known relatedness degree $r_{g_\Theta (i),j}$. When $g_\Theta (i) = \emptyset$, we instead sample $s_{i,j}$ from $p_\emptyset (s_{i,j})$, a background distribution over the SK value given an unknown pair of individuals. These conditional distributions can be readily estimated in advance based on an auxiliary dataset that includes related individuals or theoretically derived based on a population genetic model (e.g., [31]). Thus, we consider them fixed during the inference described in the following.

#### Expectation-maximization (EM) formulation for maximum likelihood estimation

Our probabilistic model induces a distribution over *S*, conditioned on $\Theta$ and *R*. For simplicity, we view $\Theta$ as model parameters without a prior, and *R* as latent variables with an input prior. We adopt the Expectation-Maximization (EM) formulation for optimizing $\Theta$ with respect to the *expected log-likelihood* of the model in the presence of unobserved variables in *R*, defined by the optimization problem

$$\begin{aligned} \text {maximize}_\Theta \ \ L(\Theta ; S) := \mathbb {E}_{R}[\log p(S,R;\Theta )], \end{aligned}$$

where $\Theta$ is subject to the constraints of a valid mapping, i.e., unique assignment of each haplotype to an individual and inclusion of at most two haplotypes per individual for each chromosome. A local optimum for this problem can be obtained by alternating between two optimization steps: (1) an E-step, where the posterior distribution over *R* in iteration *t*, denoted $p(R|S;\Theta ^{(t)})$, is computed based on the current parameter estimates $\Theta ^{(t)}$; and (2) an M-step, where updated parameters $\Theta ^{(t+1)}$ are obtained by maximizing the expected log-likelihood

$$\begin{aligned} \mathbb {E}_{R\sim p(R|S;\Theta ^{(t)})}[\log p(S,R;\Theta ^{(t+1)})], \end{aligned}$$

where the expectation is taken with respect to the posterior estimated in the previous E-step. It can be shown that this procedure returns a non-decreasing objective after every update and converges to a local optimum [32].

#### Belief updates for relatedness profiles (E-step)

We first describe the computation of $p(R|S;\Theta )$ given $\Theta$. Recall that $\Theta$ defines a mapping $g_\Theta$ from reconstructed haplotypes to the target individuals. The posterior belief over each $r_{j,\ell }$ can be computed separately as follows:

$$\begin{aligned} p(r_{j,\ell } | S,\Theta ) \propto p(r_{j,\ell }) \prod _{i \in g_\Theta ^{-1}(j)} p(s_{i,\ell } | r_{j,\ell }). \end{aligned}$$

In other words, the distribution over $r_{j,\ell }$ is proportional to its prior probability multiplied by the product of probabilities of generating the observed SK values between each haplotype assigned to individual *j* given by the current linkage $\Theta$, including all chromosomes, and individual $\ell$ in the relative set.

#### Minimum-cost network flow algorithm for linkage optimization (M-step)

The M-step represents a combinatorial problem of matching haplotypes to individuals that maximize the likelihood of the given probabilistic model. We introduce a network flow algorithm to efficiently perform this optimization. First, note that the expected log-likelihood objective can be expanded as

$$\begin{aligned} \sum \limits _{i\in [N]} \left[ \left( \sum \limits _{j\in [T]} \theta _{i,j}\cdot \sum \limits _{\ell \in [M]} \mathbb {E}_{r_{j,\ell }\sim p(r_{j,\ell }|S ;\Theta )}\left[ \log p(s_{i,\ell } | r_{j,\ell }) \right] \right) + \theta _{i,T+1} \cdot \sum \limits _{\ell \in [M]} p_{\emptyset } (s_{i,\ell }) \right] , \end{aligned}$$

which is a linear combination of the model parameters $\Theta$. To further simplify, given the output of the E-step, i.e., $p(r_{j,\ell }|S ;\Theta )$, consider precomputing the following quantity

$$\begin{aligned} \pi _{i,j} := \left\{ \begin{array}{ll} \sum _{\ell \in [M]} \mathbb {E}_{r_{j,\ell }\sim p(r_{j,\ell }|S ;\Theta )}\left[ \log p(s_{i,\ell } | r_{j,\ell }) \right] &{} \text {if }j\in [T], \\ \sum _{\ell \in [M]} p_{\emptyset } (s_{i,\ell }) &{} \text {if }j=T+1, \end{array}\right. \end{aligned}$$

for each $i\in [N]$ and $j\in [T+1]$. Then, the optimization problem for the M-step can be alternatively expressed as the integer linear program

$$\begin{aligned} \text {maximize}_\Theta{} & {} \ \ \ \ \sum \limits _{i\in [N]} \sum \limits _{j\in [T+1]} \theta _{i,j} \pi _{i,j}, \\ \text {subject to}{} & {} \ \ \ \ \theta _{i,j}\in \{0,1\}, \ \ \ \forall i\in [N], j\in [T+1], \\{} & {} \ \ \ \ \sum \limits _{j\in [T+1]} \theta _{i,j} = 1, \ \ \ \forall i\in [N], \\{} & {} \ \ \ \ \sum \limits _{i\in [N]} {\textbf {1}}\{ \textsf {chr}(i) = c \} \cdot \theta _{i,j} \le 2, \ \ \ \forall j\in [T], c\in [22], \end{aligned}$$

where $\textsf {chr}(i)$ indicates the chromosome of the reconstructed haplotype (considering only 22 autosomes for simplicity).

We solve this problem using a minimum-cost network flow algorithm as follows and as illustrated in Additional file 1: Fig. S9B. We introduce *N* source nodes (one for each reconstructed haplotype), each with a supply of 1, and 22 sets of $T+1$ sink nodes (one set per chromosome), each with a maximum capacity of 2, except for the last sink node of each set, which represents no match (hence not subjected to a capacity constraint). Each source node is connected to all $T+1$ sink nodes in the set corresponding to the chromosome of haplotype *i* (i.e., $\textsf {chr}(i)$). The cost of each edge between the source *i* and the sink *j* (in the corresponding chromosome set) is set to $-\pi _{i,j}$. It is straightforward to see that the minimum-cost flow on this graph corresponds to a solution to the above problem with the integer domain constraint ($\theta _{i,j}\in \{0,1\}$) removed as a relaxation. The amount of flow on each edge in the solution between the source *i* and the sink *j* (in the corresponding chromosome set) is taken as $\theta _{i,j}$. Once all EM iterations have been completed, we finally round the solution to obtain an approximate integer solution; we note that in our experiments the optimal solution always coincided with an integer solution.

#### Construction of an initial solution

Here, we describe the preliminary steps used to generate an initial linking prediction to be input into the algorithm.

##### Step 1: Chaining adjacent chunks by extending the sequence via imputation

We first attempt to form short chains of adjacent chromosome chunks from the same individual by imputing the target set chunks against the relative set and trying to match the imputed dosages with the genotypes of reconstructed haplotypes in adjacent chunks. More specifically, we do the following: (1) for each chunk, we try to “extend” it by imputing (using minimac4) against the relative set over a region that extends one chunk in each direction (where possible; for a chunk at the start or end of a chromosome, the region only extends in one direction). (2) For each boundary between chunks, we consider a window extending 500 Kb in each direction and, for each pair of target set chunks across the boundary, compute a score which attempts to capture their likelihood of being from the same individual. This score is computed by comparing the actual genotypes of a chunk with the imputed dosages of the adjacent chunk in the pair at every variant in the window, as the number of variants for which the dosage difference is above some threshold (we use 0.5). (3) At each boundary, pairs of chunks are linked if they are each other’s sole closest match (by minimum score) and their score is sufficiently low (we use $\le$ 5). (4) Chains are formed by simply joining linked pairs with a chunk in common.

##### Step 2: Grouping chunks with identical relatedness patterns

We separately form groups of target set chunks in the following way: we first threshold the SK values at a fixed constant $1/(2^{9/2})$ and group haplotypes that have identical relatedness patterns with respect to the individuals in the relative set, discarding any sets smaller than 5 haplotypes. Then, we threshold the SK values of the ungrouped haplotypes again at a lower value of $1/(2^{13/2})$, adding to existing groups or creating new ones based on the relatedness pattern, and again discard sets smaller than 5 haplotypes. (The higher and lower thresholds above correspond to expected lower bounds for 3rd and 5th degree relationships, respectively [33].) Groups are capped to include at most two haplotypes per chunk, as the intent is to predict the haplotypes of a diploid individual. This capping is executed for each group by finding the relative with the strongest relationship by SK value to any haplotype in the group, then selecting up to two haplotypes per chunk with the highest SK value with that particular relative.

##### Step 3: Combining the solutions from steps 1 and 2

We finally combine the information from the two steps above to get an initial prediction to be input into the linking algorithm, in the following way: the chunks in a chain formed in step 1 above are assigned to a group (representing a single individual of origin) if more than half of the chunks in the chain are in a corresponding group produced by step 2. We are, in effect, connecting chains formed in the imputation step using SK information.

#### Implementation details

We implement the EM algorithm described above, using the NetworkX Python package [34] for the network flow implementation of the M-step. We initialize $\Theta$ before the initial E-step using the preliminary steps described above in the “Construction of an initial solution” section.

We set *T* to the number of individuals represented in the target set; a precise value of *T* is not required in practice, as long as it is sufficiently large, because our algorithm includes a no-match state which allows some of the target individuals to have an empty set in the output if the quality of the match is not high.

We estimate the conditional distribution over the SK values for each relatedness degree, including the background distribution, based on a global set of related pairs of individuals from our dataset. While in a real attack scenario these distributions would be estimated from an independent source of information, we adopted this approach due to a lack of another large dataset with a sufficient number of relatives; note that these distributions are not expected to be dataset-specific and are instead based on the fundamental properties of the recombination process. To further establish that the algorithm could successfully leverage SK distributions based on an external dataset, we conducted an additional experiment in which we split our dataset sample-wise into two equal-sized, non-overlapping groups, estimating distributions from one group and running the haplotype linking algorithm on the other. We found that the algorithm performed with comparable success (Additional file 1: Fig. S19).

### Haplotype linking experiments

#### Data

We tested our haplotype linking program using genetic data from 2,000 AoU participants, including 100 pairs of related individuals (20 pairs each of 1st through 5th degree relatives) and randomly sampled 900 pairs of unrelated individuals. For the primary linking experiment with chunks, all 44 haploid autosomal chromosomes from one individual in each pair were split into chunks using a chunk length of 20M base positions and included in a target set (for a total of 310,000 haploid chunks, shuffled to obscure the individual of origin), with the other individual in each pair assigned to the relative set. For the additional experiment linking full chromosomes, the only difference is that the target set consisted of chromosome-length haplotypes that were not further divided into chunks.

#### Computing SK scores

To prepare the GERMLINE2 input, the target set and relative set samples were combined into a single file. GERMLINE2 was run on this input file in haploid mode (option -h), with a minimum match length of 2.5 cM (option -m), and with a minimum minor allele frequency of 0.01 (option -f). Other options were left in their default settings.

#### Inference algorithm settings

The initial prediction taken as input by the algorithm was produced by the preliminary steps described above (see the “Construction of an initial solution” section). See Additional file 1: Fig. S10 for the results of step 1 and Additional file 1: Fig. S11 for the results of steps 2 and 3. Since step 1 produced very little error, combining it with the output of step 2 resulted in an initial prediction with relatively few errors (compared to the output of step 2 alone) to provide as input to the linking algorithm.

The target haplotypes were first filtered to reduce the size of the linking problem to only consider the haplotypes related (by SK threshold $1/(2^{13/2})$) to a relative set sample related to other target haplotypes. The prior probability $p(r = \emptyset )$ that a pair of individuals is unrelated was set to 0.9, with the remaining probability divided uniformly among the other degrees. For conditional distributions over SK values for different degrees of relatedness, we used empirical distributions in the form of histograms with 25 equal-sized bins spanning (0, 1], plus an additional bin at zero. We used a combined distribution to represent the 3rd, 4th, and 5th degree relationships together. For the distribution over SK values given an unrelated pair, we used a point mass at zero set to a probability of 0.95, plus a Gaussian distribution at the empirical mean and with adjustable standard deviation set to 0.1. Our results for this experiment are based on a single iteration of the algorithm, as further iterations did not lead to significant changes in the solution. Only linked sets of at least five haplotypes were included in the output.

For the chromosome linking experiment (Additional file 1: Fig. S12), step 1 of the preliminary linking steps (see the “Construction of an initial solution” section) was not relevant, so the initial prediction taken as input by the algorithm was the output of step 2. The target haplotypes were first filtered in the same way as in the chunk linking experiment. The same prior was used for relatedness degrees. For the conditional distributions over SK values for the different relatedness degrees, we used Gaussian distributions set to the empirical means and standard deviations (here based on chromosome-wise rather than chunk-wise SK values), after observing that the distribution of SK values more closely follow a Gaussian at the level of chromosomes. As before, we used a combined distribution to represent the 3rd, 4th, and 5th degree relationships together. For the distribution over SK values given an unrelated pair, we used a Gaussian distribution at the empirical mean and with adjustable standard deviation set to 0.0175, which led to a better balance between correctly linking haplotypes while minimizing the number of errors in this setting. We ran the algorithm for 10 iterations on initial groups including at least 10 haplotypes as input, and reported only linked sets of at least five haplotypes that show significant relatedness to at least one relative set sample ($p(r = \emptyset ) \le 0.99$).

### Theoretical analysis of haplotype linking practicality

In this part of our work, we sought to evaluate how plausible it is that an adversary would have access to the relatives required for haplotype linking to be effective. To accomplish this, we used a population genetic model (see details below), which is essentially an extension of the one used by Erlich et al. [14], to estimate the probability that a given reference panel individual has relatives of different degrees in some external genomic database (Additional file 1: Fig. S13). We then combined these estimates with our results from the haplotype linking experiment to quantify the proportion of reference panel genomes an adversary might be able to reconstruct with access to a genomic database of a particular size.

#### Population genetic model

What we aimed to obtain is, for each relationship degree 1st- through 5th-degree, the probability of one or more detectable relatives of a target, of that degree, being in a database. For any degree including more than one type of relationship, this is approximated by a sum of probabilities, one for each relationship.

We consider relatives for each of these degrees to be only up to one generation apart. For example, a target’s grandaunt or granduncle is a 3rd-degree relative but is two generations from the target, so this type of 3rd-degree relationship is not accounted for. We believe this to be reasonable because databases leveraged for linking are likely to be relatively contemporary with the target, given the newness of most genetic databases, and therefore relatives more than one generation apart would be relatively uncommon. This would make these omitted probabilities quite low. In addition, the inclusion of these probabilities would only strengthen our estimates of the number of relatives that would be available to an attacker. Potentially, an attacker’s ability to leverage relatives a greater number of generations apart will increase over time, as current databases become dated. All this said, it is straightforward to extend our model to consider a wider range of relationships.

The assumptions and derivations of our model are based on the work of Erlich et al. on identity inference using long-range familial search [14], in which the authors explored a similar question of how many relatives of a person can be expected in a public genealogy database. Our model differs by the addition of probability formulas for each particular degree of relative, 1st through 5th degree, and by the inclusion of particular relationships (siblings, parent-child) not accounted for by the generalized formulas in their model.

The assumptions of the model are as follows:

1. The model is a monogamous Wright-Fisher model [35, 36]. The current generation of the population consists of *N* males and *N* females, paired into *N* couples. Each individual in the current generation draws its parental couple randomly from the couples in the previous generation.
2. Each couple has *r* offspring (2.5 in our analysis), so the size of the population *g* generations before the current one is $N(g) = N(r/2)^{-g}$.
3. We consider only diploid individuals and the autosomal chromosomes.
4. The database contains the data of *R* total individuals from the model population, all of whom are from the most recent generation and the previous one, represented in proportion to the relative sizes of those generations.
5. A target individual belongs to the most recent generation. This means, for example, that hypothetical cousins once removed of the target in a younger generation are not accounted for-they are assumed not to exist, given the Wright-Fisher assumption and the target being in the current generation. This simplifying assumption is motivated by the fact that the auxiliary database available to an attacker is likely to have been collected earlier than the up-to-date reference panels typically leveraged by imputation servers.
6. The target individual’s genome is compared to those of all *R* individuals in the database, and IBD segments are detected from these comparisons. It is assumed that IBD segments must have length at least *m* Morgans in order to be detectable. And for a relationship to be determined with confidence, at least *s* segments must be detected. In our analysis, $m = 0.06$ (6cM) and $s = 2$.

For the full model derivations, see Additional file 1: Supplementary Note 2.

#### Applying the model to estimate the probability of a relative match in an auxiliary database

In calculating actual probabilities for relatives of the different degrees, we used the same values for *m*, *s*, and *r* as did Erlich et al.: $m = 0.06$ (6 cM), $s = 2$, and $r = 2.5$ (as noted above, *m* is the minimum detectable length in Morgans of an IBD segment, *s* is the minimum number of segments that must be detected in order to determine a relationship with confidence, and *r* is the number of offspring produced by each couple in a generation). The probabilities actually depend only on the *proportion* of the population in the external database relative to the overall population, not on the precise sizes of the population (*N*) and database (*R*) independently, so in the implementation we set the proportion directly with a range of possible values. The resulting probabilities for different proportions are plotted in Additional file 1: Fig. S13.

We note that the model must be applied with caveats, given its simplifying assumptions. For example, inherent in the Wright-Fisher model are the assumptions of a homogeneous population and discrete non-overlapping generations. In addition, when we apply our formulas to estimate the proportion of reference panel individuals who would have relatives in an external database, we are assuming that the reference panel individuals come from the same population as the external database individuals to which they are compared. While our theoretical analysis is likely to deviate from reality to an extent dependent upon the precise attack scenario and databases that are available to an attacker, we view our analysis as establishing a plausible evidence, based on a standard population genetic model, for the success of haplotype linking strategy.

#### Computing the empirical distribution of haplotype linking performance

In order to estimate the proportion of individuals and their genomes we would expect an adversary to be able to link given an *n*th-degree relative (Fig. 3B), we began with the linked set data produced by our haplotype linking experiment, which we translated (for each degree) into an empirical distribution over proportion of genome linked. Note that here proportion of genome is in terms of the base pair (bp) lengths of the linked chunks, which we believe is more appropriate for quantifying the reconstructed genome than the *number* of linked chunks. We then generated a smoothed probability density function (PDF) estimation from each degree’s empirical distribution using the package bde [37] for bounded density estimation (we used the “jonesCorrectionMuller94BoundaryKernel” function). Each smoothed PDF was converted into a CDF, which represents the probability of linking genome proportion $\ge g$. The curves in Fig. 3B show the probability value of the CDF on the *x*-axis, with the corresponding genome proportion on the y-axis, and each point (*p*, *g*) on the curve indicates that at least proportion *g* of the genome can be linked for proportion *p* of the samples according to our empirical estimates.

#### Synthesizing model and empirical results to estimate linking performance in practice

 To summarize an adversary’s ability to perform haplotype linking with recovered reference panel haplotypes (Fig. 3C), we combined our model probabilities (Additional file 1: Fig. S13) and the smoothed curves described above (Fig. 3B) as follows. First, assuming independence of the model probabilities for different degrees, we computed for each degree the probability of a reference panel individual having a *closest* relative of that degree in the relative set (so as not to double-count individuals with more than one relative). Then, we calculated the *expected* proportion of the reference panel having a closest relative of each degree, which is equal to the probability of a single reference panel individual having a closest relative of that degree, assuming independence among individuals. To obtain the curves in Fig. 3C, we then computed, for each genome proportion *g* (on the *y*-axis), the total proportion of samples (on the *x*-axis) for which more than *g* of genome could be linked according to our previous empirical estimates. This is achieved by (i) retrieving the sample proportion for each degree corresponding to the threshold *g* from the curves shown in Fig. 3B, (ii) multiplying these values by the expected proportion of the reference panel having a closest relative of each degree (which converts the values to proportions of the entire reference panel), and (iii) summing the resulting sample proportions across degrees. We repeated this calculation for several different relative set sizes, i.e., considering relative sets including different proportions of the underlying population.

### Supplementary information

**Additional file 1.** Supplementary Information.

**Additional file 2.** Review history.

## Data Availability

The 1000 Genomes dataset is publicly available from the International Genome Sample Resource (IGSR) at: https://www.internationalgenome.org/. The All of Us (AoU) Controlled Tier Dataset v5 is available through the Controlled Tier of the AoU Researcher Workbench. The application to access the AoU dataset can be submitted at: https://www.researchallofus.org/register/. Our code is available in Zenodo, where, due to the sensitive nature of our study, it is stored in a controlled-access repository: https://doi.org/10.5281/zenodo.10000981 [38]. We will share access to our code with anyone affiliated with an academic or research organization who provides a valid rationale for needing access. We consider valid rationale to include use for ethical academic research purposes and to specifically not include use against public imputation servers. If you would like access, please use the Zenodo link above to submit a request including (1) your affiliation, (2) a brief description of the purposes for which you are requesting access, and (3) a statement affirming that you will use the code only for research purposes and will not use it against existing imputation servers or attempt to reconstruct data to which you do not have direct access. We expect to be able to review and approve requests within 2–3 weeks.
