# Supplementary material for: Reconstruction of private genomes through reference-based genotype imputation
Source: Genome Biol. 2023 Dec 6;24:271. doi: 10.1186/s13059-023-03105-6 (PMC10698978; doi:10.1186/s13059-023-03105-6)
Supplement: Supplementary file 1 — Additional file 1. Supplementary Information. [file 13059_2023_3105_MOESM1_ESM.pdf]

Supplementary Information

Reconstruction of private genomes through reference-based genotype imputation

Contents

Supplementary Figures

Figure S1: Example queries and dosage outputs

Figure S2: Empirical dosage distribution of imputation output by query match cardinality

Figure S3: Perfect reconstruction when imputing queries with unique matches

Figure S4: Distribution of number of mismatches in reconstructed haplotypes

Figure S5: Reconstruction performance vs. reference panel size

Figure S6: Reconstruction performance vs. reference panel ancestry composition

Figure S7: Haplotype reconstruction against imputation algorithms other than minimac

Figure S8: Haplotype reconstruction performance on a sub-chromosome chunk

Figure S9: Graphical illustration of our haplotype linking algorithm

Figure S10: Preliminary chunk linking using imputation

Figure S11: Preliminary chunk linking using semi-kinship (SK) information

Figure S12: Haplotype linking performance on chromosome-length haplotypes

Figure S13: Probability of a relative match in a database based on a population genetic model

Figure S14: Leveraging GEDmatch for haplotype linking

Figure S15: Haplotype reconstruction running time

Figure S16: Haplotype linking running time and computational requirements

Figure S17: Haplotype reconstruction on MAF-filtered reference panels

Figure S18: Query match cardinality classifier accuracy

Figure S19: Haplotype linking with independently estimated SK coefficient prior distributions

Figure S20: Examples of misclassified queries and their dosage output

Supplementary Notes

Supplementary Note 1: Analysis of reconstruction performance differences across reference panels

Supplementary Note 2: Population genetic model derivations

Supplementary Figures

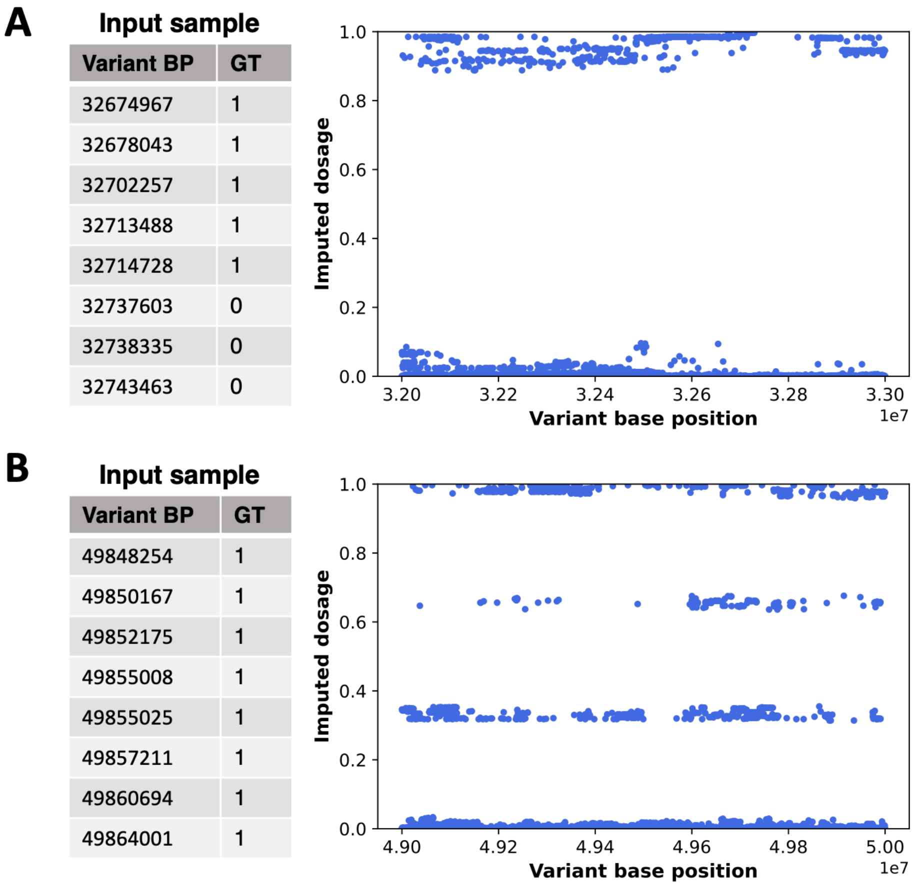

Figure S1. Example queries and dosage outputs. Each of (**A**) and (**B**) shows an example of a query sequence of 8 variants on chromosome 20 which exactly matches one or a small number of haplotypes in 1000 Genomes Phase 3 (1KG), and a segment of the imputation dosage output resulting from the query. Each query is represented in terms of variant base positions (BPs) and corresponding genotype assignments (GTs), with 0 representing the REF allele and 1 representing the ALT allele. The query shown in (**A**) perfectly matches only one 1KG haplotype, belonging to sample HG01859. Observe the extreme dosage values in the window of dosages shown. The genotypes in the output in fact match one of HG01859's haplotypes along the entire length of chromosome 20, with no mistakes. The query shown in (**B**) matches 3 reference panel haplotypes. Notice the dosages concentrated around multiples of 1/3, which is typical for a query with 3 matches.

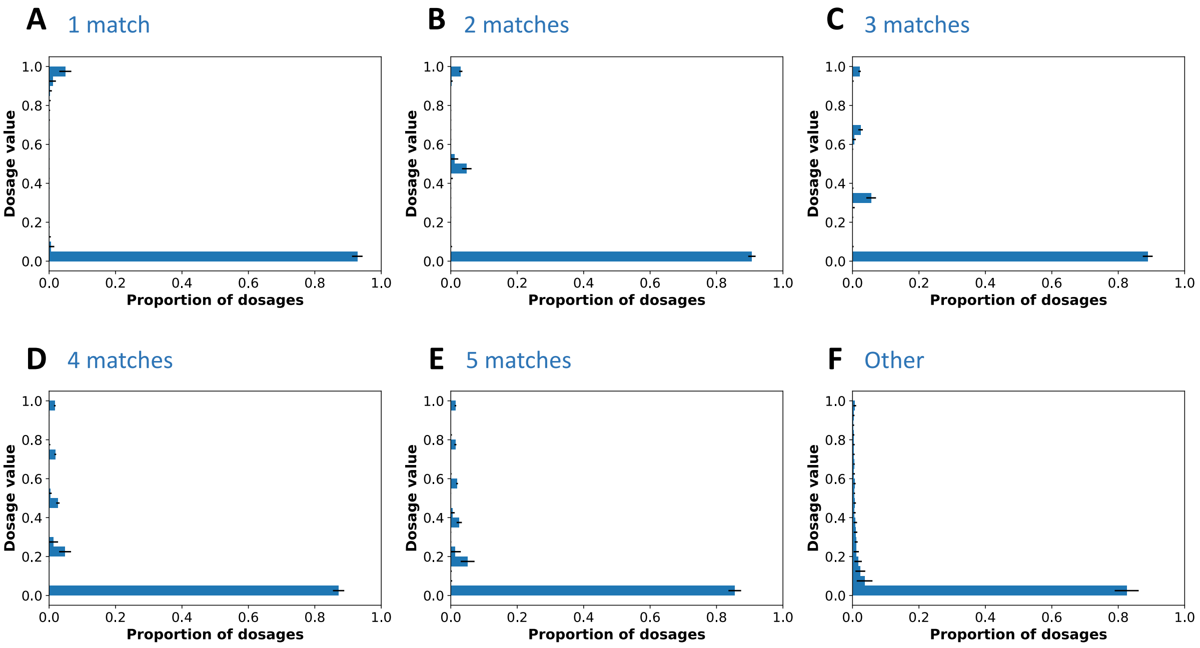

Figure S2. Empirical dosage distribution of imputation output by query match cardinality. Shown are observed distributions of dosage values in the output of imputation (with 1KG reference panel, chromosome 20) run on queries exactly matching different numbers of reference panel haplotypes: one through five (**A**, **B**, **C**, **D**, **E**), and more than five or none (Other; **F**). Each plot represents the proportions of dosage values falling in 20 equal-sized bins covering the range [0, 1], averaged over 100 sets of imputation output on queries of the corresponding match cardinality type. These are the same data used to train the random forest classifier used in haplotype reconstruction. The distinct patterns of different query types allow the classifier to accurately predict the match cardinality of a query based only on the imputation output. An error bar represents the standard deviation of the dosage proportion in a bin over the 100 sets.

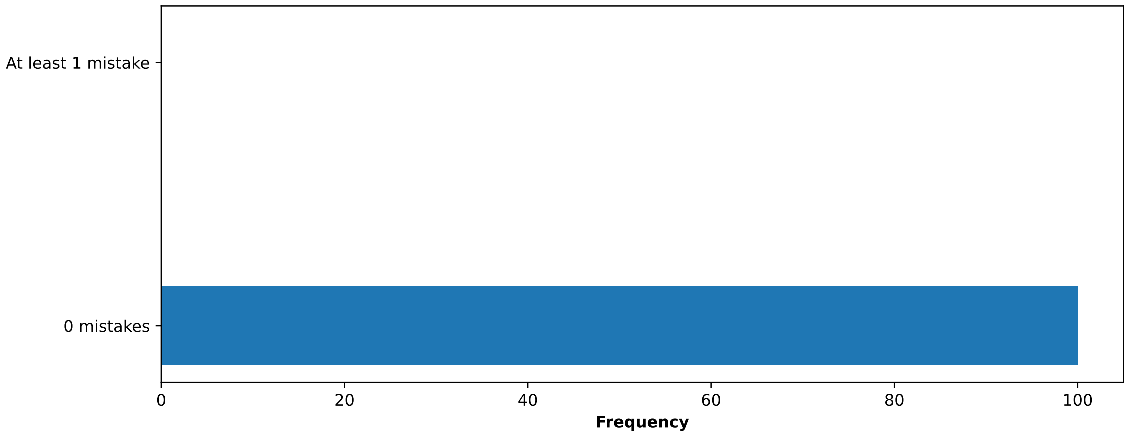

Figure S3. Perfect reconstruction when imputing queries with unique matches. One hundred queries, among those constructed as described in the Methods section and each matching exactly one 1KG chromosome 20 reference panel haplotype, were imputed against the 1KG panel and each one's output was compared to its closest haplotype match in the panel. The number of mistakes (mismatching variant-level genotypes) compared to their closest matches are shown. Every query resulted in the perfect reconstruction of a reference panel haplotype over the whole length of the chromosome.

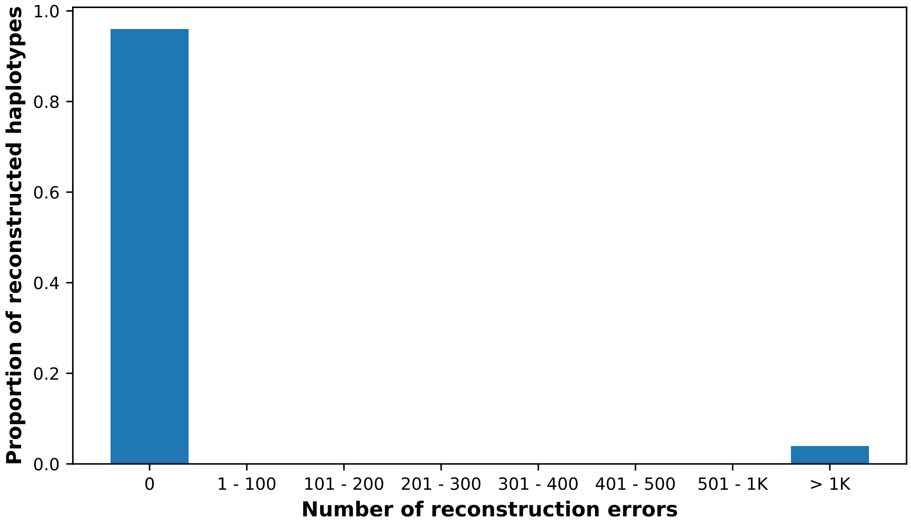

Figure S4. Distribution of number of mismatches in reconstructed haplotypes. We show a histogram of error counts for all haplotypes obtained from the 1KG reference panel in one million queries in the haplotype reconstruction experiment represented in **Fig. 2A**. This includes redundantly constructed haplotypes. For each haplotype obtained in the experiment, its error count is considered to be the number of variant allele differences (mismatches) compared to its closest haplotype match in the reference panel. The vast majority of haplotypes reconstructed by our program either exactly matched a reference panel haplotype (0 mistakes) or had more than 1,000 mistakes. In **Fig. 2**, we chose to define a "correct" reconstruction as having 100 mistakes or fewer, but any reasonably small number in the range of 1 to 500 would have produced similar plots.

A

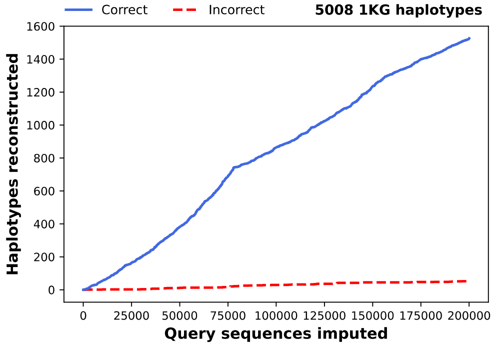

Actual num. query matches \ Predicted num. query matches	1	2	3	4	5	Other	Sum
1	2208	0	1	0	0	259	2468
2	1	681	0	0	8	142	832
3	0	0	495	0	0	58	553
4	0	0	0	354	0	93	447
5	0	0	0	0	135	27	162
Other 6+	28	2	205	51	3	737	1026
Other 0	29	42	49	30	2	194360	194512
Sum	2266	725	750	435	148	195676	200000

B

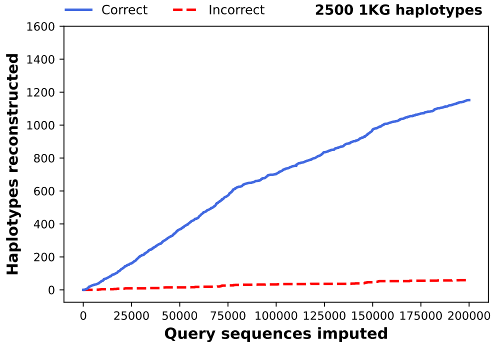

Actual num. query matches \ Predicted num. query matches	1	2	3	4	5	Other	Sum
1	2095	0	1	2	0	338	2436
2	3	669	0	1	8	177	858
3	0	0	449	0	0	74	523
4	0	0	0	274	0	91	365
5	0	0	0	0	109	30	139
Other 6+	13	0	104	22	0	312	451
Other 0	67	96	137	76	18	194834	195228
Sum	2178	765	691	375	135	195856	200000

C

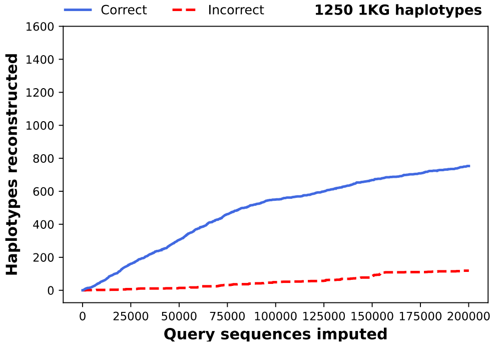

Actual num. query matches \ Predicted num. query matches	1	2	3	4	5	Other	Sum
1	1842	0	0	0	0	328	2170
2	0	544	0	0	4	131	679
3	0	0	330	0	0	53	383
4	0	0	0	176	0	40	216
5	0	0	0	0	55	18	73
Other 6+	4	0	30	6	0	90	130
Other 0	167	212	136	128	31	195675	196349
Sum	2013	756	496	310	90	196335	200000

Figure S5. Reconstruction performance vs. reference panel size. Haplotype reconstruction was run using as reference panel: (**A**) full 1KG (2504 samples; 5008 haplotypes), (**B**) a random subsample of 1KG of 1250 samples (2500 haplotypes), and (**C**) a random subsample of 1KG of 625 samples (1250 haplotypes). For each experiment, we plotted the trajectory of reconstruction progress (left) and recorded the number of queries associated with each pair of ground truth match cardinality type and the predicted type from the classifier (right). Note that the "Other" type includes both 0 matches and 6 or more matches, and the classifier does not distinguish between the two cases. A reconstructed haplotype was "correct" if it had no more than 100 genotype differences from a RP haplotype and that closest haplotype had not been reconstructed correctly previously. The count of "incorrect" haplotypes was incremented if a reconstructed haplotype had more than 100 genotype differences from the closest RP match and was sufficiently different (>100 mismatches) from the previous incorrect haplotypes. Holding constant the population from which the samples are drawn allows us to better examine the effect of panel size. As panel size increases, correct reconstruction rate increases and query misclassification rate decreases. We further discuss these effects in **Supplementary Note 1**.

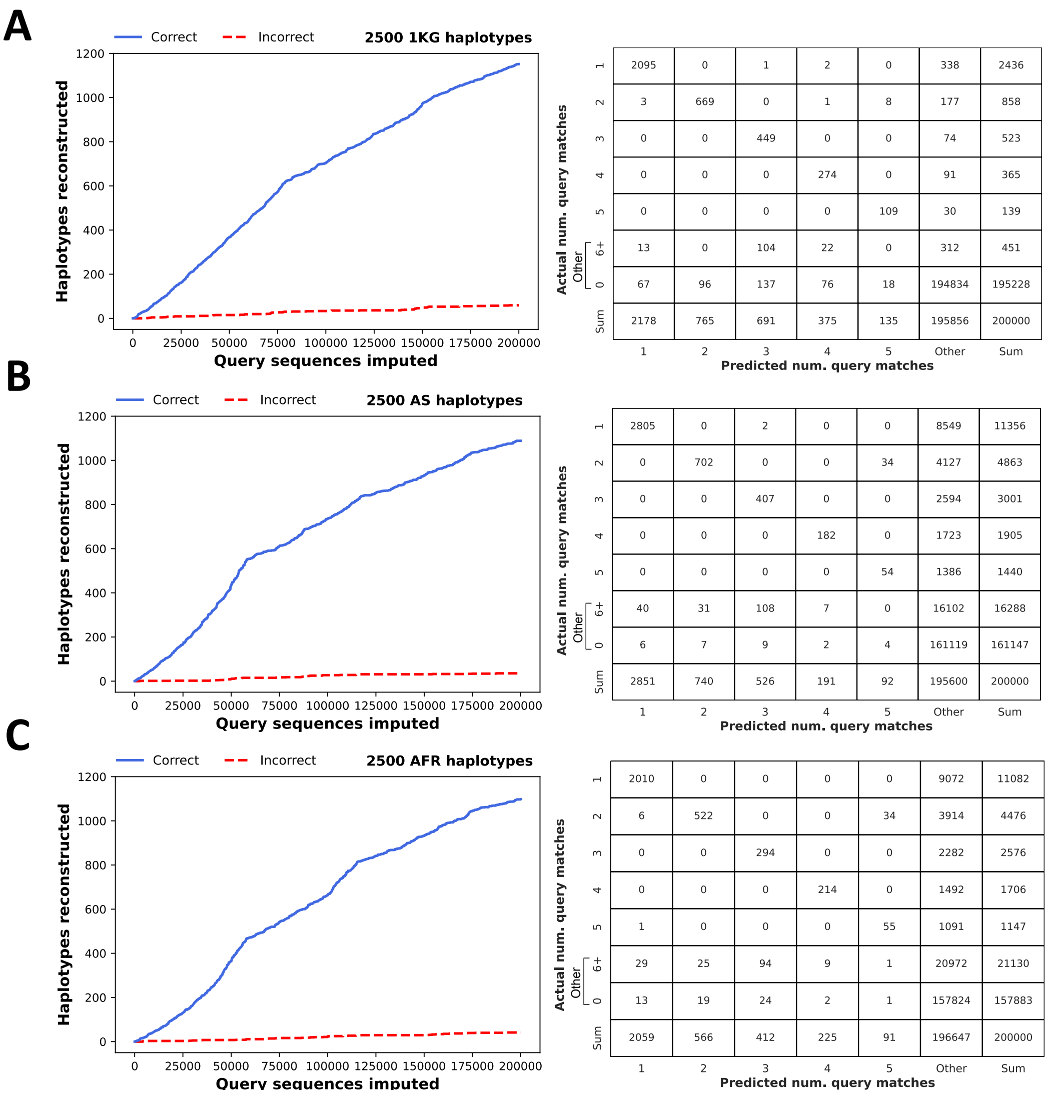

Figure S6. Reconstruction performance vs. reference panel ancestry composition. Haplotype reconstruction was run using as reference panel: (**A**) a random subsample of 1000 Genomes Phase 3 (1KG) of 1250 samples (2500 haplotypes), (**B**) the full All of Us Asian American (AS) panel used in our **Fig. 2** experiments (1250 samples), and (**C**) a random 1250-sample subset of the All of Us Black or African American (AFR) used in our **Fig. 2** experiments. For each experiment, we plotted the trajectory of reconstruction progress (left) and recorded the number of queries associated with each pair of ground truth match cardinality type and the predicted type from the classifier (right). Note that the "Other" type includes both 0 matches and 6 or more matches, and the classifier does not distinguish between the two cases. A reconstructed haplotype was "correct" if it had no more than 100 genotype differences from a RP haplotype and that closest haplotype had not been reconstructed correctly previously. The count of "incorrect" haplotypes was incremented if a reconstructed haplotype had more than 100 genotype differences from the closest RP match and was sufficiently different (>100 mismatches) from the previous incorrect haplotypes. Holding panel size constant allows us to better examine the effect of panel ancestry composition. Although the reconstruction curves look quite similar, the underlying query matching and classification patterns are different, indicating an interplay of different factors. We further discuss these effects in **Supplementary Note 1**.

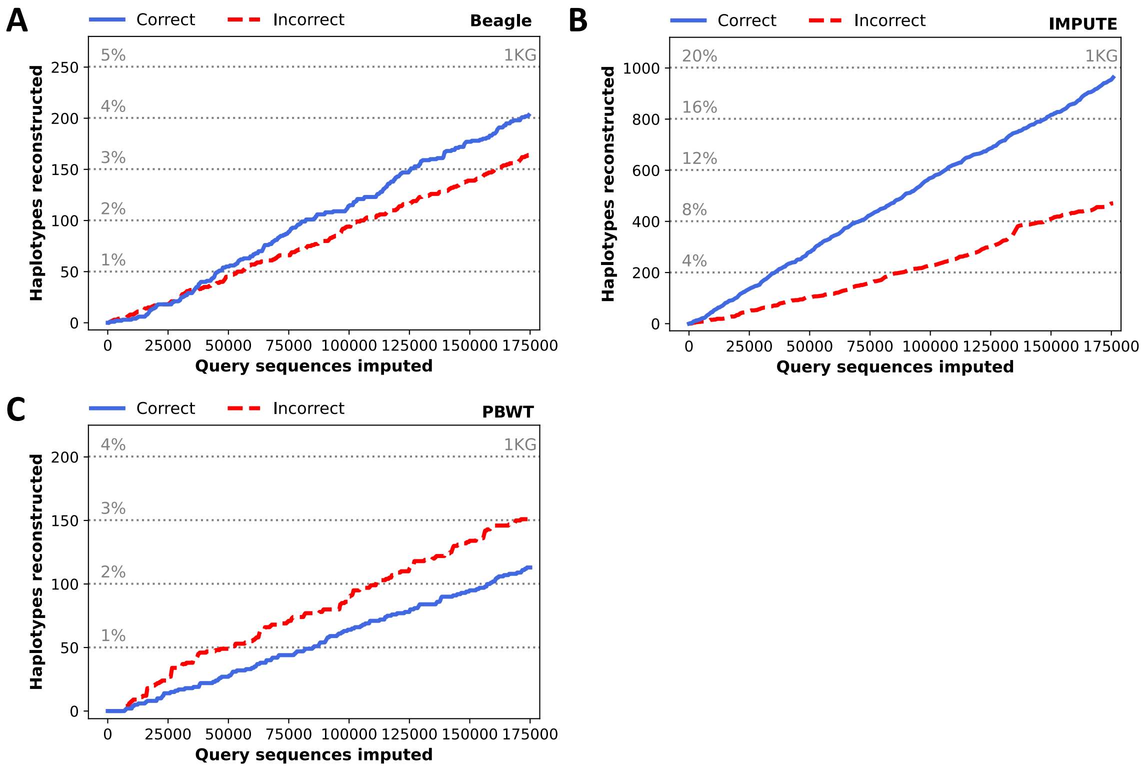

Figure S7. Haplotype reconstruction against imputation algorithms other than minimac. The "discrete-genotype" version of haplotype reconstruction, using only 0/1 genotype predictions from imputation and not continuous dosage data, was run with 1000 Genomes Phase 3 (1KG) chromosome 20 as the reference panel and (**A**) Beagle5.4, (**B**) IMPUTE5, and (**C**) PBWT for the imputation algorithm. The results indicate different rates of reconstruction for the different algorithms, but in all cases, reconstruction is demonstrated to be feasible with sufficient accuracy. A reconstructed haplotype was "correct" if it had no more than 100 genotype differences from a RP haplotype and that closest haplotype had not been reconstructed correctly previously. The count of "incorrect" haplotypes was incremented if a reconstructed haplotype had more than 100 genotype differences from the closest RP match and was sufficiently different (>100 mismatches) from the previous incorrect haplotypes. Horizontal dotted lines represent percentages of the total number of haplotypes in a RP. Note that the y-axes differ in scale.

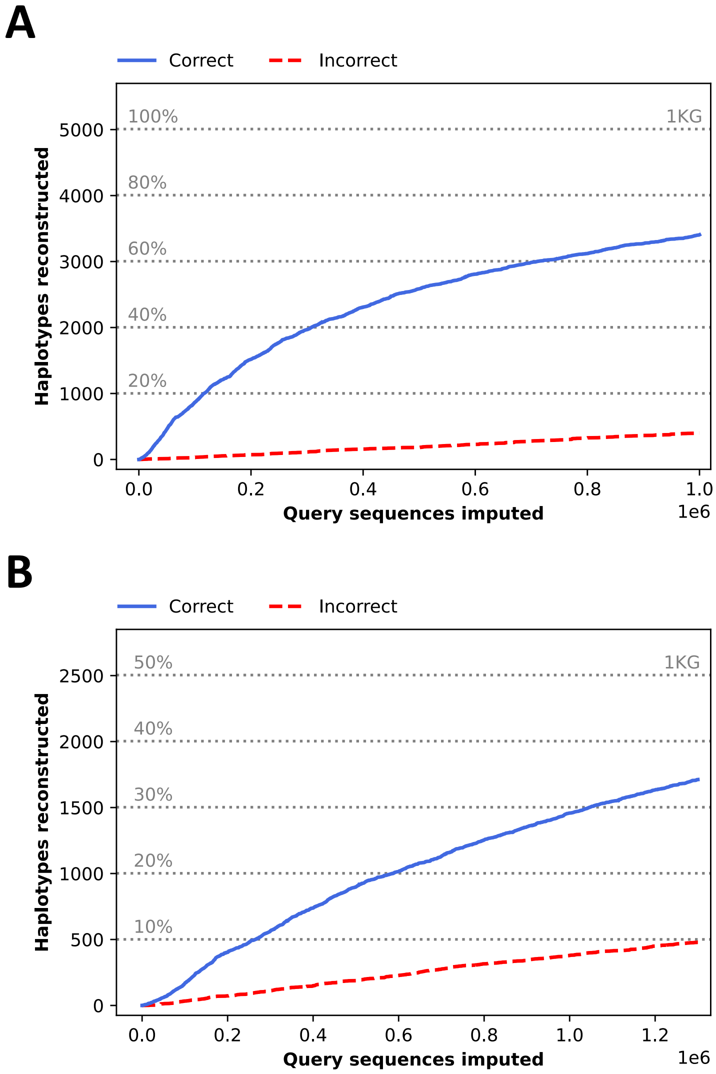

Figure S8. Haplotype reconstruction performance on a sub-chromosome chunk. Haplotype reconstruction based on (**A**) fractional (expected) imputed dosages and (**B**) discrete genotype predictions were run with the first 20-Mbp chunk of chromosome 20 of 1KG as the reference panel, using minimac4 for imputation. The results are similar to those for reconstructing full chromosomes in **Fig. 2**, but reconstruction occurred here with a slightly lower rate of reconstruction. A reconstructed haplotype was "correct" if it had no more than 100 genotype differences from a RP haplotype and that closest haplotype had not been reconstructed correctly previously. The count of "incorrect" haplotypes was incremented if a reconstructed haplotype had more than 100 genotype differences from the closest RP match and was sufficiently different (>100 mismatches) from the previous incorrect haplotypes. Horizontal dotted lines represent percentages of the total number of haplotypes in a RP.

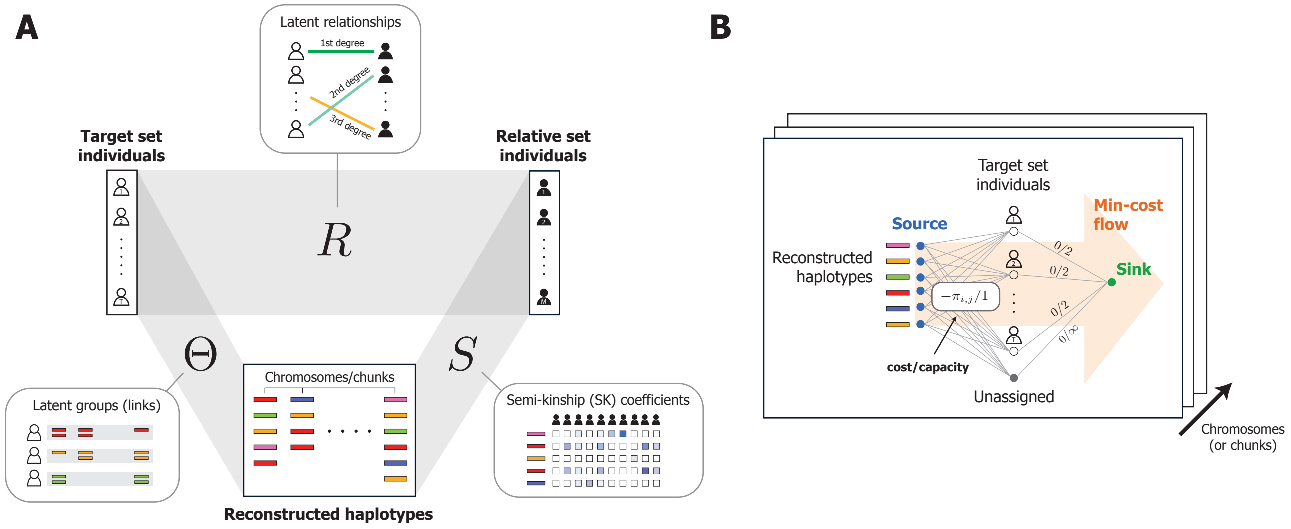

Figure S9. Graphical illustration of our haplotype linking algorithm. We depict the key components of our probabilistic model of haplotype linking (**A**) and the flow network used for the minimum-cost flow component of our algorithm (**B**). As shown in (**A**), the main variables of the problem include: Θ, representing the assignment of input haplotypes to target individuals; R, representing the degree of relatedness between each target individual and relative set individual; and S, the observed semi-kinship coefficients between the input haplotypes and relative set individuals. Note that the linking problem is to infer Θ based on S, where R is also unknown. The network in (**B**) is constructed separately for each chromosome or chunk being considered for linking. Each input haplotype is a source node with an input flow of 1, which flows through the network to reach the sink node. The cost (a) and capacity (b) of each edge is indicated as a/b. In the intermediate layer of the network, each target set individual is represented by a node, whose incoming flow determines which haplotypes are assigned to that individual. The cost of assignment is set to $-\pi_{i,j}$, defined in **Methods**, which creates the equivalence between the min-cost flow problem on this network and the optimization of Θ in the M-step of our linking algorithm. Note the outgoing capacity of 2 for each intermediate node, which ensures no more than two haplotypes are linked to the same individual for each (diploid) chromosome or chunk. An extra intermediate node (Unassigned) with infinite capacity serves as a sink for input haplotypes that could not be assigned to an existing group. See **Methods** for additional algorithm details.

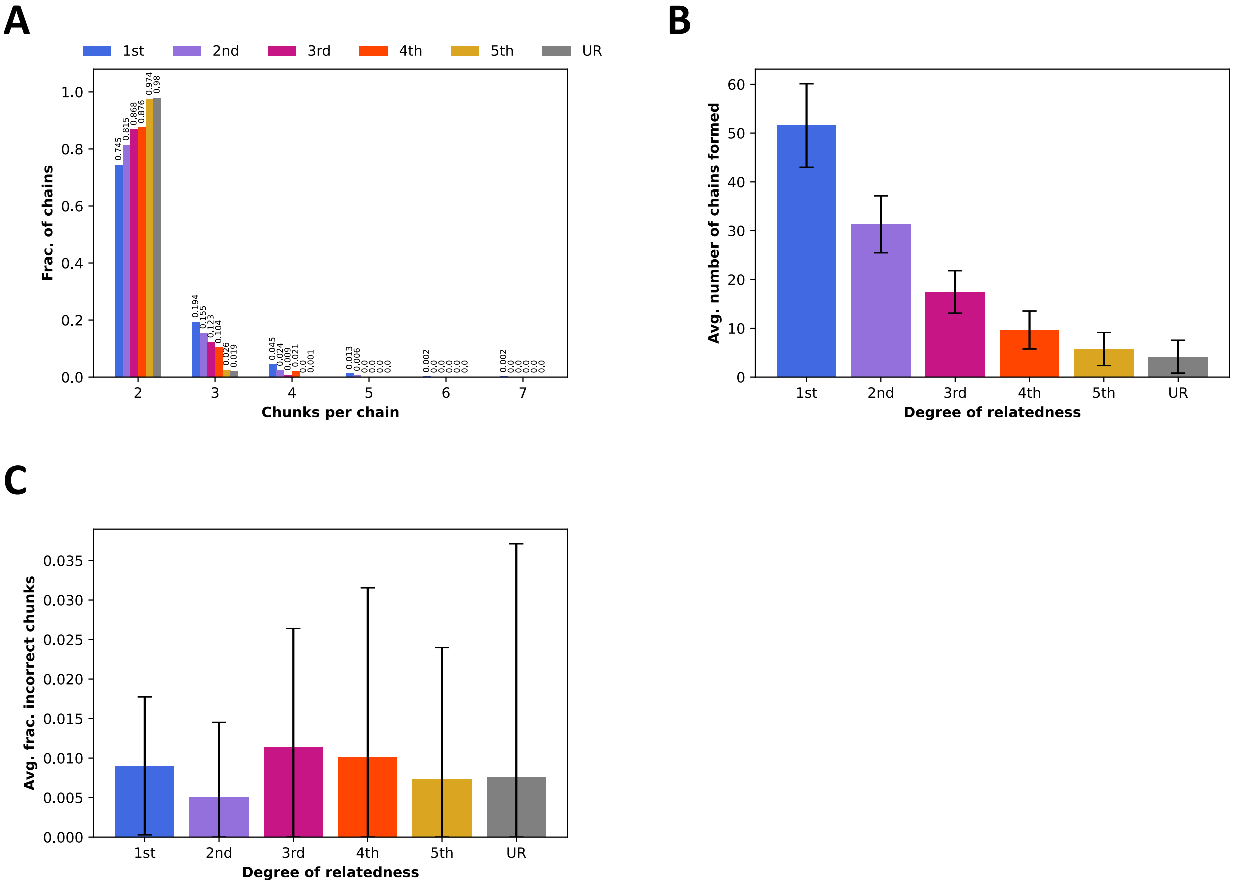

Figure S10. Preliminary chunk linking using imputation. As a preliminary step used to generate an initial linking prediction (to be input into the linking algorithm), minimac4 was used to impute all chunks against the relative set and the output was leveraged to form links between adjacent chunks where possible (as described in **Methods**, in Step 1 of "Construction of an initial solution"). The results of this process are chains of consecutive linked chunks predicted to correspond to the same individual. Note that each chain can only be composed of chunks within the same chromosome. (**A**) The distribution of chain length, by degree of the relative the individual to which the chain chunks correspond has in the relative set. Unrelated (UR) individuals, without a relative in the relative set, are also represented. Note that longer chains tend to be formed for individuals with closer relatives in the relative set. (**B**) The average number of chains formed for an individual, by degree. Error bars indicate standard deviation. We observe that the chains formed for individuals with closer relatives tend to be greater in both number and size. (**C**) The average fraction of chunks among the chains formed for an individual which were incorrectly assigned to that individual, by degree. Error bars indicate standard deviation. We observe relatively low error rates across all groups for this strategy.

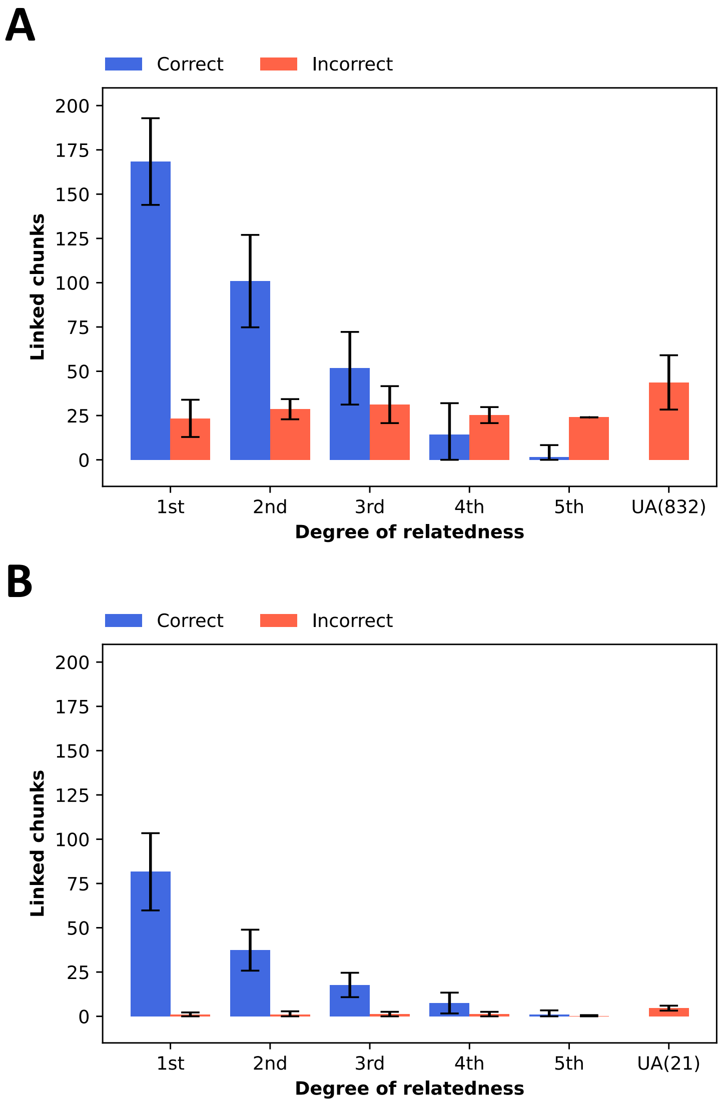

Figure S11. Preliminary chunk linking using semi-kinship (SK) information. As a preliminary step used to generate an initial linking prediction (to be input into the linking algorithm), chunk haplotypes having sufficiently high SK values with the same relative set sample(s) were grouped (as described in **Methods**, in Step 2 of "Construction of an initial solution"). The results are shown in (**A**). These results were then combined with the result of using imputation output to link chunks, shown in **Figure S10** (as described in **Methods**, in Step 3). The product of this combination is shown in (**B**). Displayed in each plot is the average number of chunks linked, by degree of available relative. Error bars indicate standard deviation. "Incorrect" haplotypes are haplotypes that were assigned to the wrong individual. The rightmost bar represents unassigned (UA) sets, not assigned because the majority of haplotypes did not come from the same individual, with the number of such sets indicated in parentheses. Note that, although in (A) we had successfully formed large groups of haplotypes, this strategy also produced substantial error and a large number of UA groups. In (B), we observe that by combining information from both imputation and SK-based preliminary linking, we are able to obtain high-quality initial groups to provide as input to the linking algorithm.

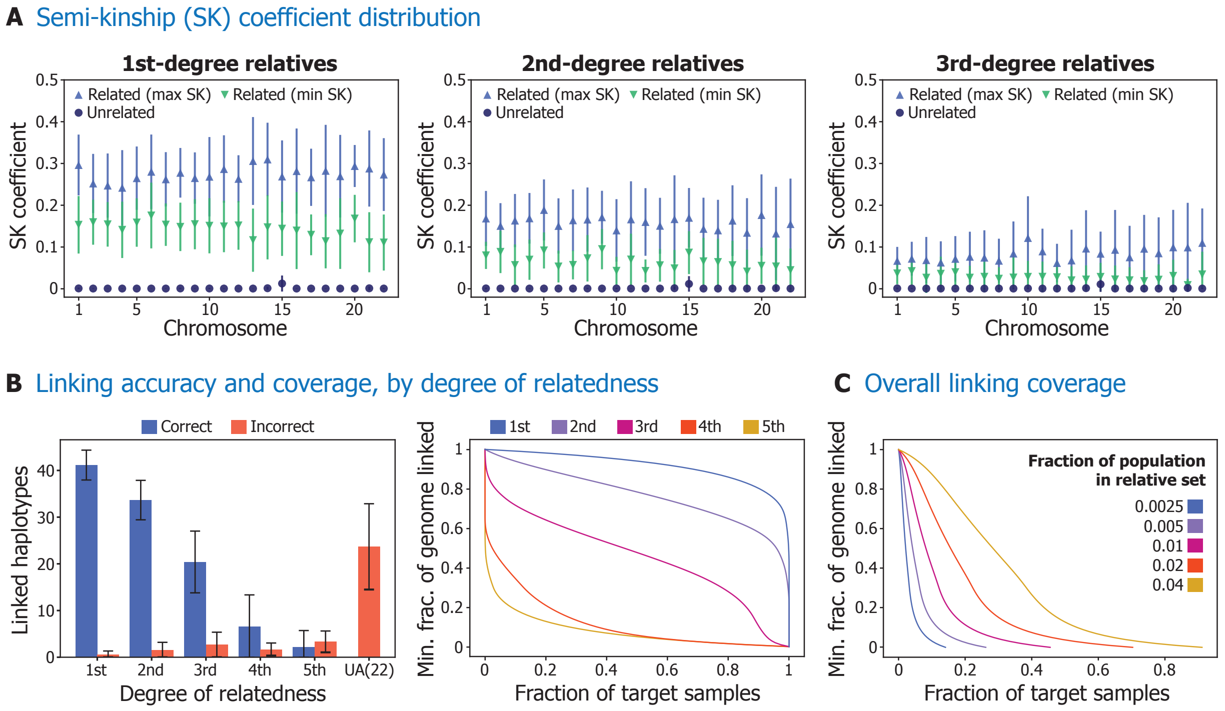

Figure S12. Haplotype linking performance on chromosome-length haplotypes. We first visualize the distribution of per-chromosome semi-kinship (SK) coefficients across different degrees of relatedness (1st, 2nd, and 3rd), compared to unrelated individuals (**A**). Markers indicate the mean, and error bars indicate standard deviation. The distributions of the larger (max) and the smaller (min) SK values between the two target haplotypes, compared against the same relative set, are plotted separately. Elevated SK for related pairs distinguishes reconstructed haplotypes from the same individual, enabling them to be linked. (**B**) Left, the average number of haplotypes linked, by degree of available relative. Error bars indicate standard deviation. "Incorrect" haplotypes refer to haplotypes assigned to the wrong individual. The rightmost bar represents unassigned (UA) sets, not assigned because the majority of haplotypes did not come from the same individual, with the number of such sets indicated in parentheses. Right, estimated proportion of individuals and their genomes (in base pair length) which an adversary could expect to successfully link, given access to an nth-degree relative for those individuals. Each point (p, g) on the curve indicates that at least proportion g of the genome could be linked for proportion p of the samples. These curves show smoothed cumulative distributions summarized in the bar chart (left). (**C**) Estimated proportion of RP individuals and the proportion of their genomes (in bp) we could expect an adversary to be able to link, given access to a relative set containing a particular fraction of the population to which the RP individuals belong. Each point (p, g) on the curve indicates that at least proportion g of the genome could be linked for proportion p of the samples. Our estimation leverages a population genetic model to calculate the probability of an adversary having access to relatives of different degrees, on which basis the degree-specific distributions in (B) are combined with weights. We observe, comparing to **Fig. 3**, that an adversary could reassemble a greater portion of reference panel genomes using chromosome-length haplotypes, compared with chunk haplotypes, given the same relative set.

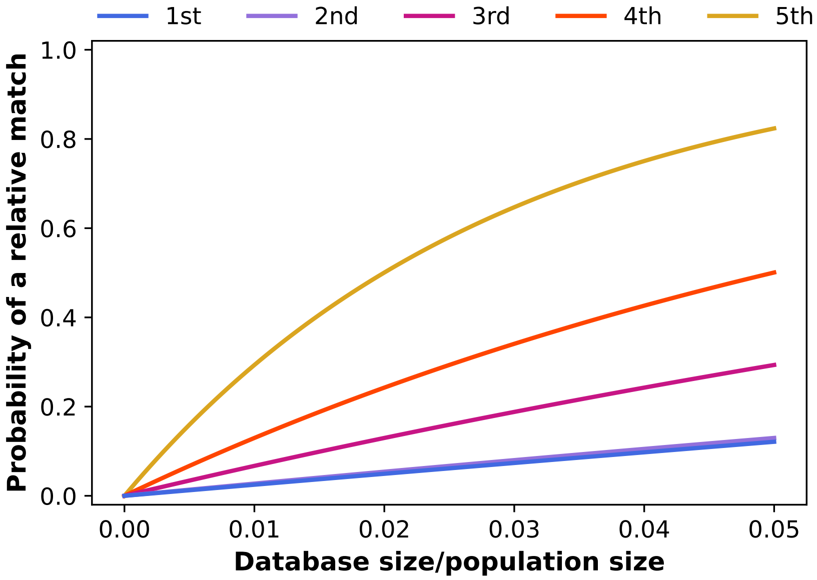

Figure S13. Probability of a relative match in a database based on a population genetic model. The probability, according to our model, of a target reference panel individual having at least one nth-degree relative in an external database, for a given size of that database. 1st-degree (e.g., sibling) through 5th-degree (e.g., second cousin) relatives are shown. Database sizes are displayed as proportion of the underlying population. The 1st- and 2nd-degree curves partially overlap and are difficult to distinguish. See **Methods** for a more detailed explanation of the model and its assumptions.

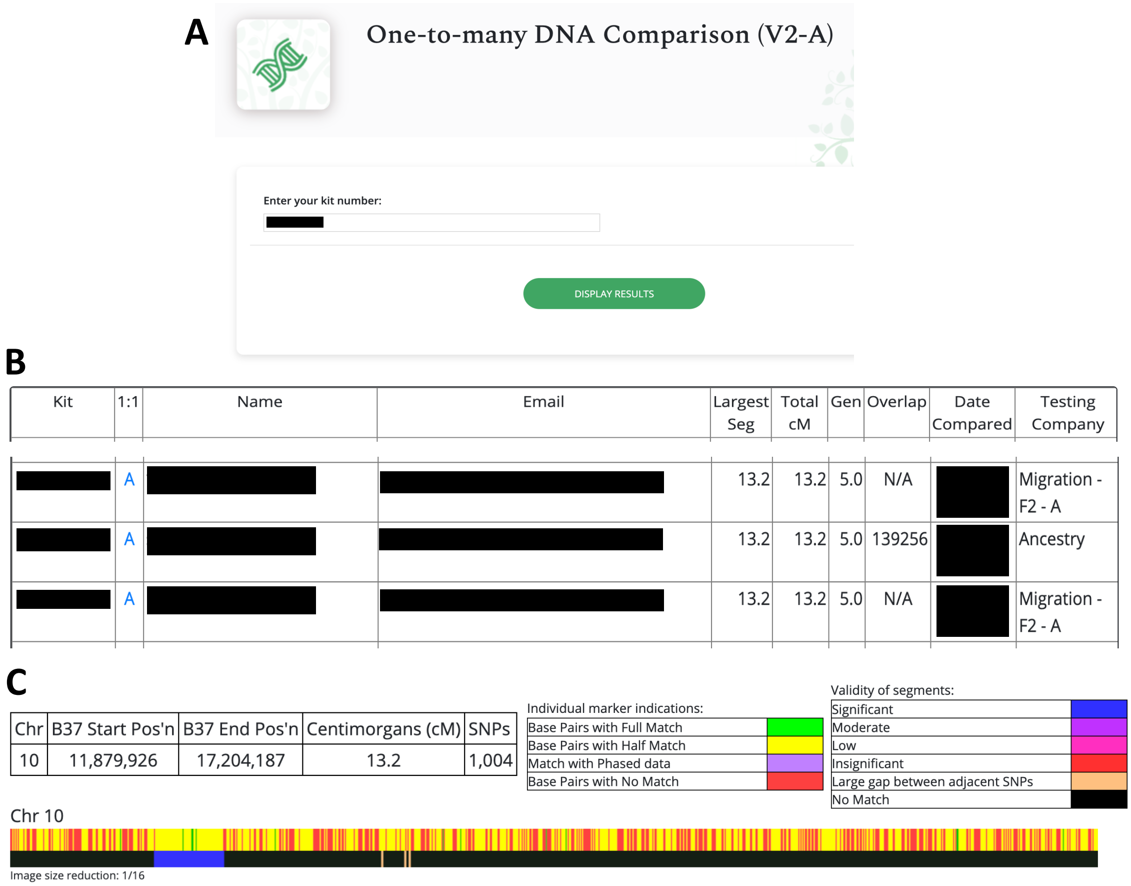

Figure S14. Leveraging GEDmatch for haplotype linking. GEDmatch users can upload their genome as a "kit" and discover relatives among the site's 1.4 million other users. An adversary attempting to link reconstructed haplotypes could leverage the site as follows: A batch of chunk haplotypes could be grouped into a full haploid artificial genome, each then duplicated to simulate diploidy, and uploaded. The one-to-many comparison tool, shown in (**A**), could then be used to see the closest matches to this kit, as in (**B**). Each closest match could be explored using the one-to-one comparison tool to see the identified IBD segments, as in (**C**), to determine which haplotype(s) the relative matched IBD. This process of uploading reconstructed haplotypes in batches and identifying their relatives could be repeated for all haplotypes, and then haplotypes related to the same GEDmatch kits could be linked, e.g., using our probabilistic linking algorithm. Users with free accounts can upload only up to 5 kits, but paying "Tier 1" users appear not to have any limit. Note that we did not upload any data to explore this workflow—anyone can make a free account and explore this given a kit ID, and it is easy to find kit IDs publicly (in a GEDmatch tutorial video, for example).

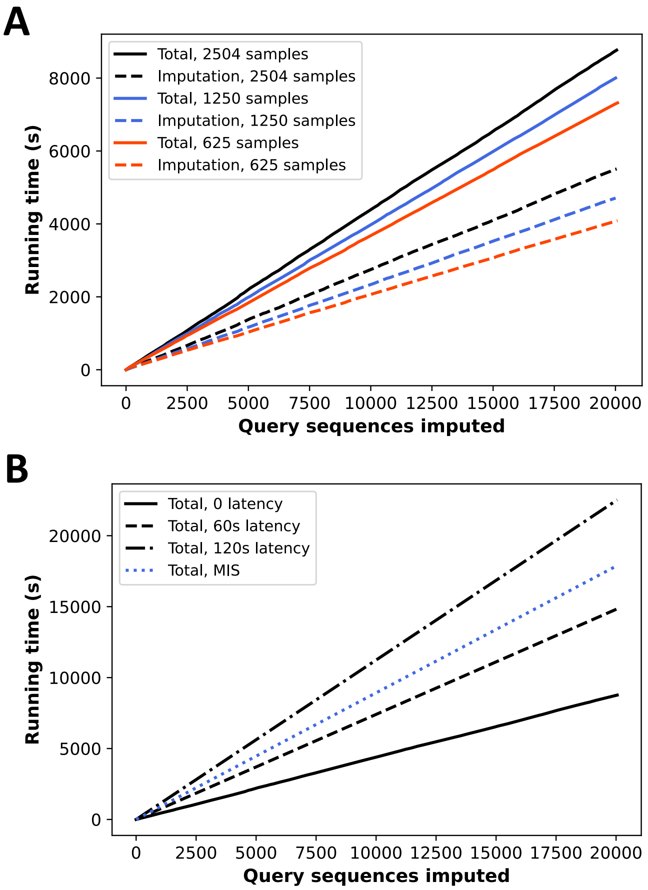

Figure S15. Haplotype reconstruction running time. (**A**) Haplotype reconstruction was run using as a reference panel the first 20-Mbp chunk of 1000 Genomes Phase 3 (1KG) chromosome 20 (2504 samples) and two random subsamples of this panel of 1250 samples and 625 samples, and running time was recorded at intervals. Imputation was performed over a chunk in order to draw clearer analogy to running on Michigan Imputation Server (MIS), which imputes on chunks separately. A single thread was used for both imputation and the reconstruction steps around it to simplify analysis. The imputation portion of the total runtime is plotted separately, which shows that a majority of runtime is spent on imputation. (**B**) We estimate running time for a reconstruction attack given different possible amounts of imputation server latency. Here, we consider latency to be the time elapsed between submission of a query file to the server and the return of results, outside of time running imputation software. As a solid line representing running time with 0 latency, we display again the 2504-sample total runtime from (**A**). We include additional runtime estimates given hypothetical amounts of imputation server latency per 128-query batch submitted to the server (60s and 120s), and include another estimate extrapolating from the actual measured total time for processing a 128-query batch on MIS against 1KG: 119 seconds per batch (note that this is total time, not latency, since we could not observe directly how long the imputation software was running). We use a 128-query batch because this is the number of queries constructed by our strategy for a single set of 8 variants. We see from these results that, while the resources and latency of the server modify the time required for a hypothetical reconstruction attack, the attack remains feasible in realistic settings. We note that per-query latency might be reduced by grouping batches of queries so more than 128 are submitted to a server together. Using multiple server accounts for the imputation portion would also reduce total running time.

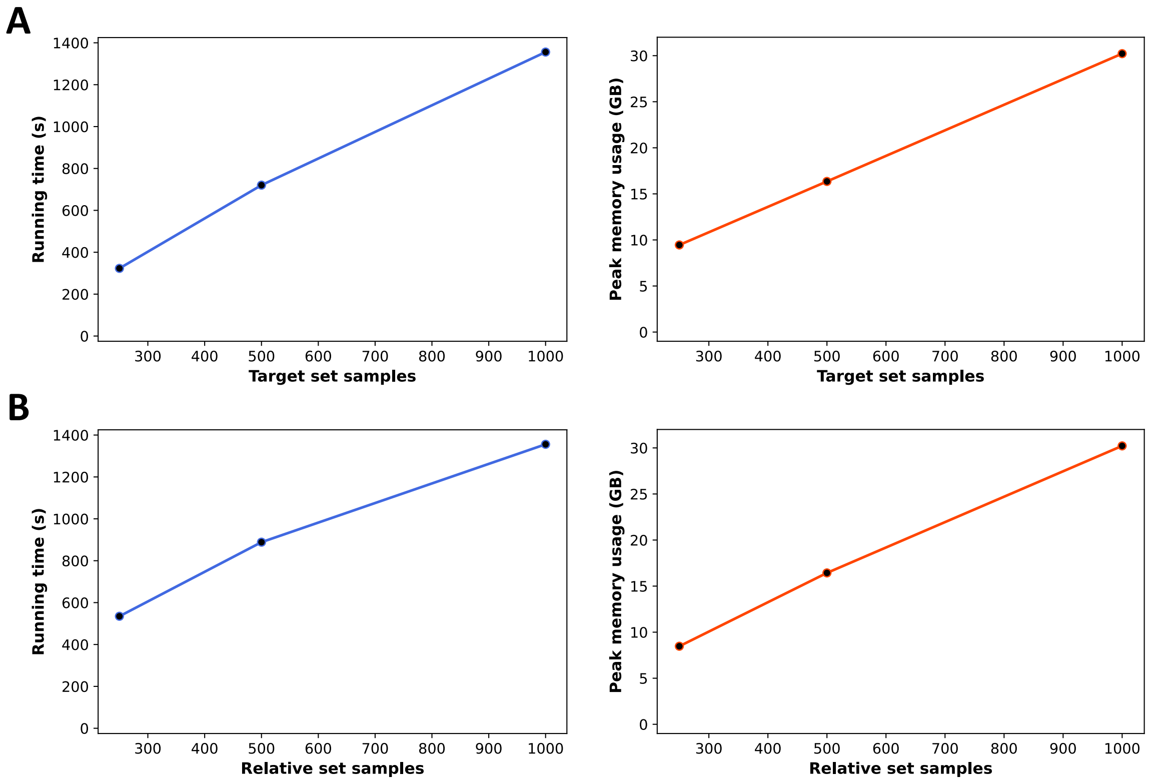

Figure S16. Haplotype linking running time and computational requirements. Shown are running time and peak memory usage for a single iteration of our probabilistic inference haplotype linking algorithm, run on subsamples of the All of Us (AoU) panels used for the primary chunk linking experiment shown in **Fig. 3**. (**A**) Both running time and peak memory usage grow roughly linearly with the number of samples included in the target set (with the relative set held fixed at 1000 samples). Note that there are 310 20-Mbp chunk haplotypes per diploid target sample in the set of haplotypes input to the linking algorithm, as in the primary experiment. (**B**) Both running time and peak memory usage grow roughly linearly with the number of samples in the relative set (with the target set held fixed at 1000 samples). Memory usage could be further reduced by processing the samples in a streaming fashion.

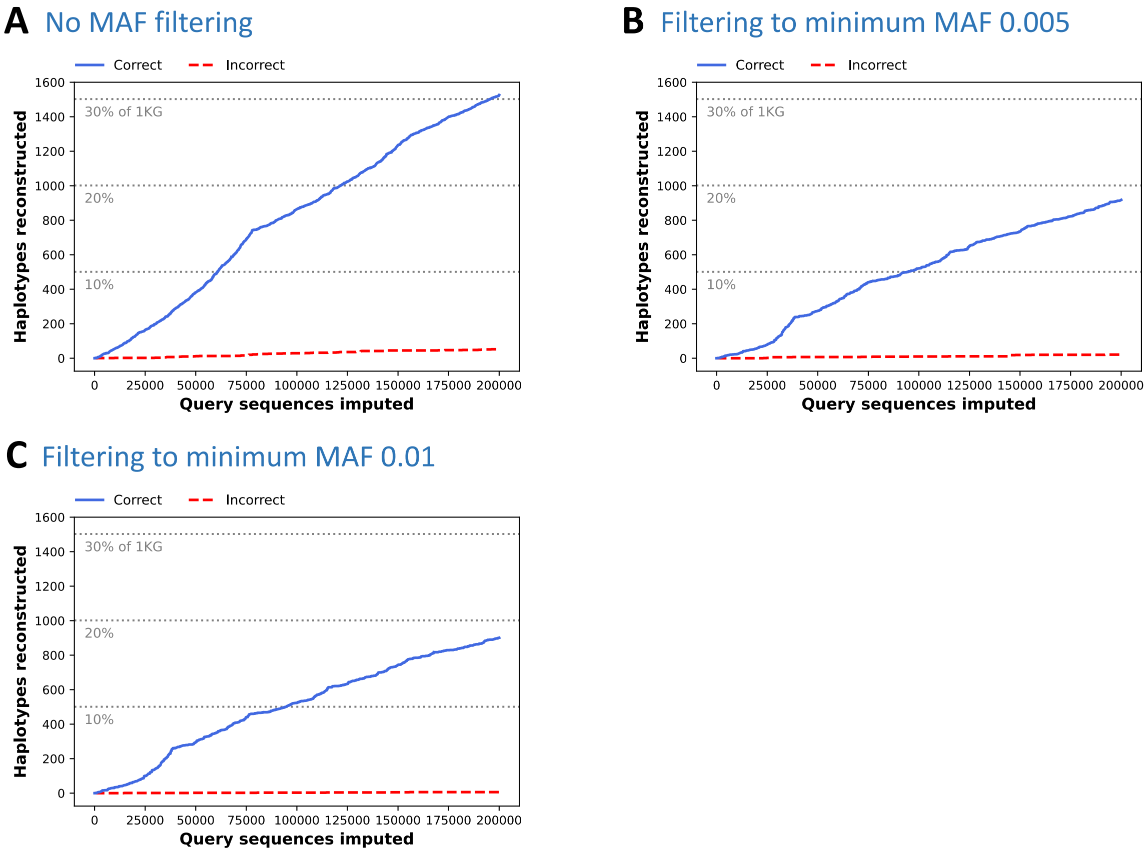

Figure S17. Haplotype reconstruction on MAF-filtered reference panels. In our primary haplotype reconstruction experiments for this study, low-minor allele frequency (MAF) variants around which queries were built were required to have a MAF of 0.005 or lower. The results of an experiment with this default setting, using 1000 Genomes Phase 3 (1KG) chromosome 20 as the reference panel, are shown in (**A**). We conducted additional experiments on the same 1KG panel in which low-MAF query variants were required to have a MAF at or below the higher threshold of 0.025 and in which variants with reference panel (RP) MAFs lower than (**B**) 0.005 or (**C**) 0.01 were ignored and not used, to simulate reconstruction on RPs filtered variant-wise by MAF. We observe from these results that filtering a RP to remove the lowest-MAF variants does slow reconstruction, but only to a limited extent. Doubling the filtering threshold from 0.005 to 0.01 has negligible additional effect, indicating that most of this protection comes from removing the very lowest-MAF variants. It is also worth noting that such a RP filtering measure would be at the cost of utility, since it may be desirable in practice to be able to impute relatively rare variants. Reconstructed haplotypes are grouped into “Correct” and “Incorrect” as defined in **Fig. 2**. Horizontal dotted lines represent percentages of the total number of haplotypes in 1KG.

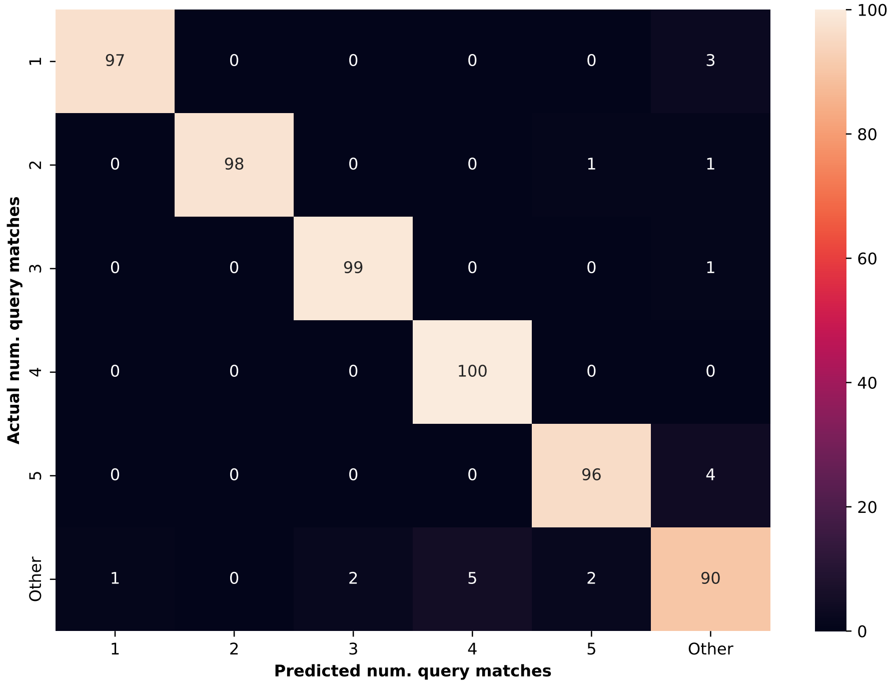

Figure S18. Query match cardinality classifier accuracy. Our random forest classifier predicts the number of reference panel haplotype matches to a query, given the dosages output when the query is imputed. The classifier was trained on 100 dosage outputs (from imputation using 1KG chromosome 20) from each of the six match-number classes (i.e., produced by a query known to have n matches): 1 through 5 and Other, where Other includes both no matches and greater than 5 matches. Here, the classifier was tested on another 100 sets of dosage data from each of the six classes. Each cell in the confusion matrix at column x and row y indicates the number of times a query which actually has y haplotype matches in the reference panel was predicted to have x haplotype matches. Additional evaluation in the setting of a full reconstruction pipeline are provided in Figures S5 and S6.

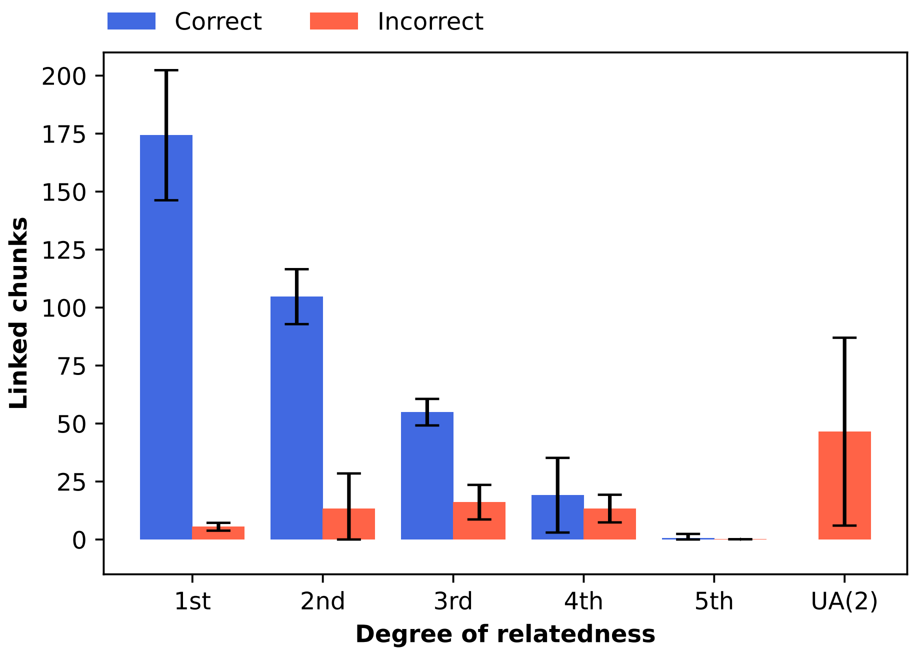

Figure S19. Haplotype linking with independently estimated SK coefficient prior distributions. The 1,000 pairs of AoU samples used in the main linking experiment shown in **Fig. 3** were divided into two groups of 500 pairs, each containing 50 pairs of related individuals (10 each of 1st- through 5th-degree relatives) and randomly sampled 450 pairs of unrelated individuals. The genetic data from one group was used to construct empirical semi-kinship (SK) distributions for the different degrees of relatedness, to be leveraged by the linking algorithm as prior distributions. Haplotype linking was tested on the other group, following the same process as the original experiment: All 44 haploid autosomal chromosomes from one individual in each pair were split into 20-Mbp chunks (310 in total) and included in the target set, for a total of 155K haplotype chunks, shuffled to obfuscate the individual of origin. The other individual in each pair was assigned to the relative set. Shown is the average number of haplotypes linked by our algorithm (out of 310 chunks in total), by degree of available relative. Error bars indicate standard deviation. "Incorrect" haplotypes refer to haplotypes assigned to the wrong individual. The rightmost bar represents unassigned (UA) sets, not assigned because the majority of haplotypes did not come from the same individual, with the number of such sets indicated in parentheses. Whereas in the original chunk linking experiment we used empirical SK distributions from the same dataset given the lack of another suitable dataset, here the distributions come from a separate held-out set of samples. We observe that this does not undermine performance of our haplotype linking algorithm, as the results here are comparable to those in **Fig. 3**.

A

Variant BP	ALT	GT
61741129	T	1
61741656	A	0
61742135	A	0
61745539	T	0
61745553	T	1
61747634	G	0
61749364	C	1
61751308	T	1

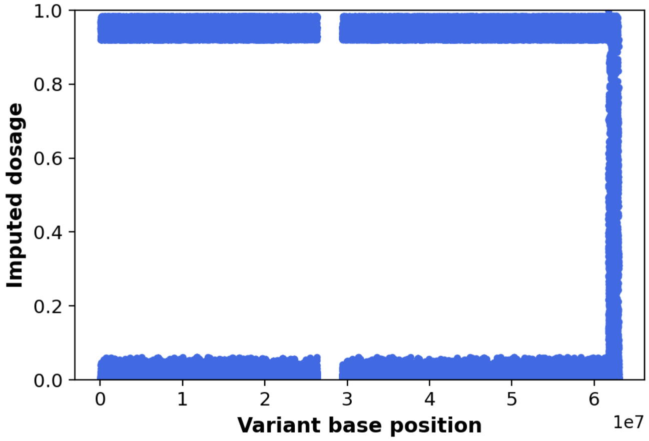

B

Variant BP	ALT	GT
55895429	C	1
55902929	C	1
55905414	A	1
55918014	G	0
55919292	G	1
55937366	T	1
55949081	C	0
55952702	A	0

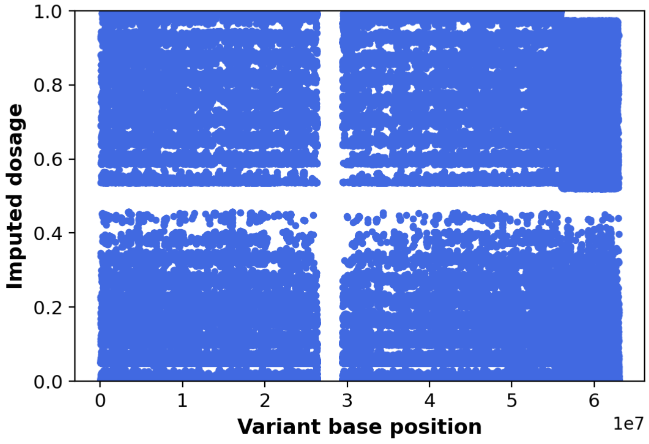

Figure S20. Examples of misclassified queries and their dosage output. Each of (**A**) and (**B**) shows a query sequence of 8 variants on chromosome 20 and its dosage output when imputed against 1000 Genomes Phase 3 (1KG). Each query is represented in terms of variant base positions (BPs), alternative allele (ALT), and corresponding genotype assignments (GTs), with 0 representing the REF allele and 1 representing the ALT allele. Dosages over the full length of chromosome 20 are shown. The query shown in (**A**) does not match any reference panel haplotypes, but was misclassified as having a single match. Observe the extreme dosage values to the left of the query's location, covering most of the window, which is similar to the pattern typical for a query with a single match and fools the classifier. This error can be prevented by restricting query construction to be closer to the middle of the chromosome. The query shown in (**B**) matches exactly one reference panel haplotype, but is assigned a prediction of "Other" by the classifier, meaning zero or greater than 5 matches. Notice that it does not display the pattern we would expect from a query with one match, which explains why it is misclassified. See **Supplementary Note 1** for a more detailed discussion of why these unexpected patterns occur and how the frequency with which they arise may vary depending on the panel used for imputation.

Supplementary Note 1: Analysis of reconstruction performance differences across reference panels

To better understand the effect of panel size on reconstruction performance, we ran additional experiments using uniformly sampled subsets of the 1KG panel of varying sizes: (1) 2504 samples (full), (2) 1250 samples, and (3) 625 samples. The results are shown in **Figure S5**. A smaller panel results in sharper curvature over the same number of queries, due to the higher rate of redundant reconstruction. We also observe that the rate of correct reconstruction grows as panel size increases, for example resulting in 381 reference haplotypes being reconstructed after 50K queries for the full panel, compared to only 305 for the smallest panel. We believe that this is likely because when a panel is larger, more queries have exactly one match (e.g., 2468 out of 200K queries for the full panel, versus only 2170 for the smallest panel) and thus result in successful reconstruction, since a greater number of different haplotypes over the query sites exist in the panel. This trend is likely to hold as panels grow larger than 1KG, at least up to a point; when the panels become large enough, the proportion of unique-matching queries may begin to decrease as more queries match more than one haplotype. In such a scenario, one could potentially design longer seeds to increase query uniqueness. Note that the vast majority of queries in our setting (e.g., 97% for full 1KG) have zero matches in the reference panel.

Next, we observed that the rate of incorrect reconstruction is inversely related to panel size. We sought to understand this pattern by stratifying the queries by their match cardinality (both real and predicted, based on our classifier). We found that when a query is correctly classified as having a single haplotype match in the reference panel, it almost always results in perfect reconstruction (see also **Figure S3**). This implies that the incorrect reconstructions are primarily an issue of *misclassification*, of queries being incorrectly predicted to have a single match.

There are two different types of such misclassifications. The first type is when a query, which actually has more than one match, is misclassified as having only one. We see from the tables in **Figure S5** that this type happens relatively infrequently (e.g., only 4 times out of 200K for the smallest panel of 1250 haplotypes), but at a slightly increased rate when the panel is larger (e.g., 29 times for the full 1KG panel), likely because when there are more haplotypes in the panel more constructed queries will match a small number of haplotypes.

The more common type of mistake occurs when a query with no perfect matches is misclassified as having one. We found that typically this occurred when a subset of genotypes on one side of the query matched a single haplotype and the query was close enough to the end of the chromosome/chunk. This could result in a unique match-looking pattern (with extreme dosage values close to 0 and 1) extending over the majority of the length of the chromosome/chunk, and confuse the classifier (see **Figure S20A** for an example). This type of mistake happens more often when the panel is smaller (e.g., 167 times out of 200K for the smallest panel and only 29 times for the largest). We hypothesize that this is because the extreme-dosages pattern will occur only when the number of nearly-matching haplotypes is small, and the number of nearly matching haplotypes is likely to be larger (resulting in a less clear pattern) when there are a greater number of haplotypes in the panel. We found that the frequency of this second type of mistake could be significantly reduced by restricting queries from being constructed too close to the ends of the chromosome/chunk. We adopted this strategy in all experiments that leverage the query classifier by only considering queries constructed in the middle 80% (in base positions) of the chromosome/chunk being reconstructed (**Methods**).

In order to better understand the effect of panel ancestry composition, we also ran experiments with the three different panels from **Figure 2**, subsampled to be the same size (1250 samples, 2500 haplotypes). The following ancestry compositions were represented: (1) the multi-ancestry 1KG population, (2) the All of Us Asian American population, and (3) the All of Us Black or African American population. The results

are shown in **Figure S6**. We first observed that the overall reconstruction performance is largely similar across the three panels. However, key differences could be seen when we examined the number of queries of different match cardinality types (both real and predicted) encountered during the reconstruction process. The two All of Us ancestry-specific panels displayed higher rates of queries that match a small number of reference panel haplotypes (e.g., more than 11,000 queries out of 200K matched exactly one haplotype for each All of Us panel, versus only 2,436 for the 1KG panel). This may be because our gnomAD MAF estimates more accurately reflect the observed allele frequencies for these ancestry-specific panels than for 1KG, which is a heterogeneous mixture of different ancestry groups. More accurate MAF estimates can boost the effectiveness of the constructed queries by encouraging the inclusion of low-MAF variants that better isolate a small number of individuals in the panel.

We also observed for the All of Us panels a much higher rate of misclassification of queries which actually match a small number of haplotypes. For example, for the Asian American panel only 2,805 out of 11,356 queries matching exactly one haplotype were correctly classified as such, and 2,010 out of 11,082 for the Black or African American panel, versus 2,095 out of 2,436 for the 1KG panel. We hypothesize that this may occur because of a greater number of nearly matching haplotypes in the All of Us panels for a typical query, since we expect the genotype patterns to be more homogeneous in ancestry-specific panels compared to 1KG. These nearly matching haplotypes can result in "fuzziness" of the imputed dosage pattern even when there is only a single exact match (see **Figure S20B**, for example). It is worth noting that, while such a misclassification is undesirable from the point of view of classification, separating it out from other unique-matching queries with the extreme-dosages patterns is desirable for reconstruction purposes, since only the latter queries can lead to accurate reconstruction regardless of the classification result.

Overall, we find that the reasons for differences in reconstruction performance across panels are complex, likely involving a range of factors such as panel size, population heterogeneity, and accuracy of MAF estimates, which together influence query uniqueness and the frequency of different error modes. Although we recognize the need to contextualize the implications of our work for different reference panels in light of these observations, we note that successful reconstruction of panel haplotypes does still occur consistently across a variety of conditions. Furthermore, the query selection strategy and classifier could likely be improved or customized (e.g., to specific populations) to further increase the correct reconstruction rate and decrease error, but such optimization was not our main goal in this study; our work focuses on demonstrating the feasibility of reference panel reconstruction in a diverse range of settings.

Supplementary Note 2: Population genetic model derivations

We begin with derivations for generalized cases and then proceed in degree order, applying the generalized formulas where possible to derive specific ones.

Generalized same-generation relatives:

Here, we consider the basic setting regarding relatives in the same generation as the target, i.e., the current generation, mainly drawing from the analysis of Erlich *et al.* [14].The goal of this derivation is to answer the following: What is the probability that a target individual has at least one detectable g-generation relative—most recent common ancestor (MRCA) g generations ago—in a database?

To begin, we note that each individual in the current generation has 2^{g-1} ancestral couples who lived g generations ago (one pair of parents, two pairs of grandparents, etc.). Assuming $2^g \ll N(g)$, the probability that a random individual in the database is a g-generation (g-gen) relative of the target, given in Eq. (2) of Erlich *et al.*'s model, is:

$$P(g\text{-gen relative}) \approx \frac{2^{2g-2}}{N(g)} \prod_{g'=1}^{g-1} \left(1 - \frac{2^{2g'-2}}{N(g')}\right), \tag{1}$$

where $N(g)$ is the size of the generation g before the current generation (see assumptions). Intuitively, this formula is the probability of sharing a MRCA g generations ago and *not* in a more recent generation.

The probability, given in Eq. (4) of Erlich *et al.*'s model, of detecting the random individual as a relative match, given that the individual is a g-gen relative of the target, can be calculated using a binomial distribution:

$$P(\text{detected} \mid g\text{-gen relative}) = 1 - \sum_{k=0}^{s-1} \text{Bin}\left(k; 2Lg + 22, \frac{e^{-2mg}}{2^{2g-2}}\right), \tag{2}$$

where where L is the length of the genome in Morgans. Here, we assume that the genome can be divided into blocks which are inherited independently. Since by definition we anticipate one crossover per Morgan of genome in expectation, and blocks are delimited by recombination or the ends of chromosomes, $2Lg + 22$ is a reasonable estimate of the number of such blocks. In each block, the probability that the target and relative share a detectable IBD segment (at least length m Morgans) is $\frac{e^{-2mg}}{2^{2g-2}}$. So intuitively, the formula subtracts from 1 the probability of $s - 1$ or fewer detectable segments (recall that at least s are needed to determine a relationship with confidence).

The probability of a given individual being detected as a g-gen relative of the target is simply the product of Eqs. (1) and (2):

$$P(g\text{-gen relative} \wedge \text{detected}) = P(g\text{-gen relative}) \cdot P(\text{detected} \mid g\text{-gen relative}). \tag{3}$$

And the probability of at least one g-generation relative (in the same generation) detected in the database (DB) is binomial:

$$P(\geq 1\ g\text{-gen relative in DB}) = 1 - \text{Bin}\left(0; \frac{R \cdot r/2}{1 + r/2}, P(g\text{-gen relative} \wedge \text{detected})\right). \tag{4}$$

Since the database contains the current and previous generations in proportion to the sizes of those generations, there are $\frac{R \cdot r/2}{1+r/2}$ individuals from the current generation in the database (as in the equation above) and $\frac{R}{1+r/2}$ from the previous generation.

Generalized cousins once removed:

Here, we consider relatives in the generation before the current one, to which the target belongs. The goal of this derivation is to answer the following: What is the probability that a target individual has at least one detectable g-generation (g-gen) relative *once removed* (1R) in a database, i.e., whose MRCA with the target is g generations before the relative and $g+1$ before the target?

The probability that a random individual in the database is a g-gen relative once removed of the target is:

$$P(g\text{-gen relative 1R}) \approx \frac{2^{2g-1}}{N(g+1)} \prod_{g'=1}^{g-1} \left(1 - \frac{2^{2g'-1}}{N(g'+1)}\right). \tag{5}$$

The formula above is a modification of Eq. (1), following the suggestion of Erlich *et al.* in their discussion of accounting for more complex relationships.

The probability of detecting an individual as a match, given that the individual is a g-gen relative once removed of the target, is:

$$P(\text{detected} \mid g\text{-gen relative 1R}) = 1 - \sum_{k=0}^{s-1} \text{Bin}\left(k; (2g+1)L + 22, \frac{e^{-(2g+1)m}}{2^{2g-1}}\right), \tag{6}$$

where $2g+1$ is the number of meioses separating the relatives. This is substituted in for the $2g$ in Eq. (2) applicable for the same-generation relative case.

Analogous to the same-generation case, the probability of an individual being detected as a g-gen relative once removed of the target is the product of Eqs. (5) and (6):

$$P(g\text{-gen relative 1R} \wedge \text{detected}) = P(g\text{-gen relative 1R}) \cdot P(\text{detected} \mid g\text{-gen relative 1R}). \tag{7}$$

And similarly, the probability of finding at least one g-gen relative once removed detected in the database (DB) is:

$$P(\geq 1\ g\text{-gen relative 1R in DB}) = 1 - \text{Bin}\left(0; \frac{R}{1+r/2}, P(g\text{-gen relative 1R} \wedge \text{detected})\right). \tag{8}$$

Next, we present the details of formula derived for each relatedness degree.

1st-degree relative:

The 1st-degree relationships we consider are: sibling-sibling and parent-child.

Sibling-sibling. Here, we derive the probability that a target individual has at least one detectable sibling in a database. For this relationship, we can apply the generalized formulas for same-generation relatives from above.

Using Eq. (1), the probability that a random individual in the database is a sibling of the target (MRCA is 1-gen ago, i.e. parents) is:

$$P(\text{MRCA is 1-gen ago}) \approx \frac{1}{N(1)}.$$

The intuition here is that the target and the random individual each have a single ancestral couple in the relevant generation, so the probability that they share the same ancestral couple is one in the number of ancestral couples in that generation.

We cannot apply Eq. (2) directly because it was derived assuming relatedness through only one side. Since siblings are related through both parents and therefore could share segments IBD on any of their 44

autosomal chromosomes, the probability of detecting a random individual as a relative match, given that the individual is the target's sibling, is:

$$P(\text{detected} \mid \text{MRCA is 1-gen ago}) = 1 - \sum_{k=0}^{s-1} \text{Bin}\left(k; 4L + 44, \frac{e^{-2m}}{2}\right).$$

Then the probability of a random individual being detected as a sibling of the target is:

$$P(\text{detected as sibling}) = P(\text{MRCA is 1-gen ago}) \cdot P(\text{detected} \mid \text{MRCA is 1-gen ago}).$$

And the probability of at least one sibling detected in the database is:

$$P(\geq 1 \text{ sibling in DB}) = 1 - \text{Bin}\left(0; \frac{R \cdot r/2}{1 + r/2}, P(\text{detected as sibling})\right). \tag{9}$$

Parent-child. Recall that we are assuming the target is in the current (most recent) generation, which implies that the only time the target has a parent-child relationship is when his or her parent is in the database. Below, we derive the probability that a target individual has at least one detectable parent in the database.

This relationship is again a special case to which our generalized formulas will not directly apply, since now the individuals do not share an ancestral couple—rather, one individual is the ancestor of the other. Still, we can take similar intermediate steps along the way to obtain the probability we desire.

The probability that a random individual in the database is a parent of the target is:

$$P(\text{parent}) \approx \frac{1}{N(1)}.$$

This is because the probability that a random individual is a parent of the target is equal to the probability that the individual is in one particular ancestral couple out of $N(1)$ in that generation.

The probability of detecting the random individual as a relative match, given that the individual is the target's parent, is:

$$P(\text{detected} \mid \text{parent}) = 1 - \sum_{k=0}^{s-1} \text{Bin}\left(k; L + 22, e^{-m}\right),$$

since there is one meiosis between parent and child, and parent and child are necessarily IBD at the blocks inherited from that parent.

Then the probability of a random individual being detected as a parent of the target is:

$$P(\text{detected as parent}) = P(\text{parent}) \cdot P(\text{detected} \mid \text{parent}).$$

And the probability of at least one parent detected in the database is:

$$P(\geq 1 \text{ parent in DB}) = 1 - \text{Bin}\left(0; \frac{R}{1 + r/2}, P(\text{detected as parent})\right). \tag{10}$$

Considering the 1st-degree case as a whole, we approximate the probability that a target individual has at least one detectable 1st-degree relative in a database as the sum of the probabilities for the sibling-sibling and parent-offspring relationships in Eqs. (9) and (10), given that these probabilities are small in realistic settings and the intersection of the two events has an even smaller probability (e.g., the product of two probabilities under independence assumption).

2nd-degree:

The only 2nd-degree relationship we need to consider is that of niece/nephew-aunt/uncle.

Neice/nephew-aunt/uncle (N/N-A/U). To derive the probability that a target individual has at least one detectable A/U in the database, we can apply the generalized formulas for cousins once removed, above. Using Eq. (5), the probability that a random individual in the database is an A/U of the target is:

$$P(\text{A/U}) \approx \frac{2}{N(2)}.$$

Next, using Eq. (6), the probability of detecting the random individual as a match, given that the individual is the target's A/U, is:

$$P(\text{detected} \mid \text{A/U}) = 1 - \sum_{k=0}^{s-1} \text{Bin}\left(k; 3L + 22, \frac{e^{-3m}}{2}\right).$$

Then the probability of a random individual being detected as an A/U of the target is:

$$P(\text{detected as A/U}) = P(\text{A/U}) \cdot P(\text{detected} \mid \text{A/U}).$$

And the probability of at least one A/U detected in the database is:

$$P(\geq 1 \text{ A/U in DB}) = 1 - \text{Bin}\left(0; \frac{R}{1 + r/2}, P(\text{detected as A/U})\right). \tag{11}$$

Since N/N-A/U is the only 2nd-degree relationship we consider, the probability of at least one 2nd-degree relative being detected in the database is the same as in Eq. (11).

3rd-degree:

The only 3rd-degree relationship we need to consider is that of first cousins.

First cousins (1C). The formulas for 1C follow from the generalized formulas for same-generation relatives, as for siblings.

$$P(\text{detected as 1C}) = \frac{4}{N(2)}\left(1 - \frac{1}{N(1)}\right)\left(1 - \sum_{k=0}^{s-1} \text{Bin}\left(k; 4L + 22, \frac{e^{-4m}}{4}\right)\right)$$

$$P(\geq 1 \text{ 1C in DB}) = 1 - \text{Bin}\left(0; \frac{R \cdot r/2}{1 + r/2}, P(\text{detected as 1C})\right) \tag{12}$$

Since 1C is the only 3rd-degree relationship we consider, the probability of at least one 3rd-degree relative being detected in the database is the same as in Eq. (12).

4th-degree:

The only 4th-degree relationship we need to consider is that of first cousins once removed.

First cousins once removed (1C1R). The formulas for first cousins once removed follow from the generalized formulas for cousins once removed, as for N/N-A/U:

$$P(\text{detected as 1C1R}) = \frac{8}{N(3)}\left(1 - \frac{2}{N(2)}\right)\left(1 - \sum_{k=0}^{s-1} \text{Bin}\left(k; 5L + 22, \frac{e^{-5m}}{8}\right)\right)$$

$$P(\geq 1 \text{ 1C1R in DB}) = 1 - \text{Bin}\left(0; \frac{R}{1 + r/2}, P(\text{detected as 1C1R})\right) \tag{13}$$

Since 1C1R is the only 4th-degree relationship we consider, the probability of at least one 4th-degree relative being detected in the database is the same as in Eq. (13).

5th-degree:

The only 5th-degree relationship we need to consider is that of second cousins.

Second cousins (2C). The formulas for 2C follow from the generalized formulas for same-generation relatives, as for siblings and 1C:

$$P(\text{detected as 2C}) = \frac{16}{N(3)} \prod_{g'=1}^{2} \left(1 - \frac{2^{2g'-2}}{N(g')}\right) \left(1 - \sum_{k=0}^{s-1} \text{Bin}\left(k; 6L+22, \frac{e^{-6m}}{16}\right)\right)$$

$$P(\geq 1 \text{ 2C in DB}) = 1 - \text{Bin}\left(0; \frac{R \cdot r/2}{1+r/2}, P(\text{detected as 2C})\right) \tag{14}$$

Since 2C is the only 5th-degree relationship we consider, the probability of at least one 5th-degree relative being detected in the database is the same as in Eq. (14).
