# Supplementary material for: Reconstruction of private genomes through reference-based genotype imputation
Source: Genome Biol. 2023 Dec 6;24:271. doi: 10.1186/s13059-023-03105-6 (PMC10698978; doi:10.1186/s13059-023-03105-6)
Supplement: Supplementary file 2 — Additional file 2. Review history. [file 13059_2023_3105_MOESM2_ESM.docx]

Review History

**First round of review**

**Reviewer 1**

**Were you able to assess all statistics in the manuscript, including the appropriateness of statistical tests used?**

Yes, and I have assessed the statistics in my report.

**Comments to author:**

It is a very interesting study to explore the risk of personal privacy leakage in the genotype imputation reference panel from an attacker's perspective. The authors focus on analyzing the latest version of the minimac imputation algorithm used by most imputation servers, including MIS and TIS, and investigate three other prominent imputation algorithms, including the PBWT algorithm used by the Sanger imputation service. The authors recommend how genotype imputation online services can mitigate the identified risks. Overall, the research is of great value in driving the balance between genomic data sharing and protecting privacy. The methods are appropriate to the study's aims, and the conclusions are adequately supported by the data shown. The work represents a significant advance and has broad interest to others in the field.

There are two comments:

1) While a series of recommendations are proposed to guide genotype imputation online services on how to mitigate privacy risks, there is a need to increase the discussion of whether suitable algorithms can ensure maximum use and avoid the possibility of privacy leakage.

2) In Figure 2, it can be seen that the results obtained from different reference panels are very different, and the authors need to analyze the internal reasons.

**Reviewer 2**

**Were you able to assess all statistics in the manuscript, including the appropriateness of statistical tests used?**

Yes, and I have assessed the statistics in my report.

**Comments to author:**

In this study, authors propose methodology to obtain reconstructed haplotypes from reference panels of public imputation servers via adversarial queries. In addition, they propose an algorithm to link the reconstructed haplotypes to form individual genomes. Imputation servers are commonly used for genomic imputation and allow researchers to impute their own genotype datasets without the need of direct access to high quality reference panels, hence detection of any possible privacy risk is of high importance for the development of adequate measures. Therefore, this work could be of broad interest to the genomics community. The manuscript is well written with extensive methodological explanation and mainly convincing (or with regard to the privacy context, concerning) results. Although I find this work interesting and valuable, there are some moderate/minor points which I believe need to be addressed.

1. The authors indicate that the imputation servers and relevant parties were informed (but in acknowledgements, they state that some servers will be notified before the publication). In what manner were they informed? Were they provided with possible protective measures indicated in the manuscript? Was there any response from these parties? Do authors think enough time was provided in case the relevant parties want to implement possible protective measures before this manuscript goes online? Although the imputation algorithms are publicly available, this work also involves server queries so I believe a green light from the relevant parties is important, especially considering the sensitivity of the subject.

2. It is stated that the minimac imputation algorithm gives extreme dosage values to the queried variants if they match exactly with one reference haplotype. What is the extent of "extreme" here? Authors show one example in Fig. S1 but it would be interesting to see the distribution of the assigned value and how much it varies with the increasing number of matches. Did authors check this or is there some reference to point to? Although the algorithm is explained in the methods, empirical or referential support for this statement could improve the manuscript since the motivation is built on it.

3. Related to the previous point, it would be useful to get a statistical measure for the authors' observations (P4: L43-48). I believe this is not a single observation?

4. Authors claim 80% of 1KG haplotypes were outputted by their reconstruction algorithm but it's not clear whether these were exact matches (P4: L53). For all the reconstructed haplotypes, do authors mean complete reconstructions? If not, what is the percentage of these? Related to this point, "correct" match was defined as having less than 100 genotype differences from the reference haplotype but authors do not justify this arbitrary choice. Overall, performance metrics need further clarification.

5. It is difficult to assess whether attack effectiveness changes with panel size with the available information (P5: L33-37). There are too many variables (like the variation between the reference panels) and figure 2 shows some difference in reconstructed haplotypes at 200K queries. In addition, there is the factor of stochasticity and the redundant outputs as the authors mention.

6. Both for the reconstruction and haplotype linking, is it possible for an adversary to obtain significance for the outputs? In other words, is there a way to assign reliability scores to the reconstructions without access to the reference panel? For haplotype linking, could the proposed population genetic model be used for this?

7. A brief discussion/information on time and computational requirements could be useful since they would be important factors for an adversarial attack.

8. Imputing rare alleles is notoriously difficult and authors' attack queries are built around a very low frequency (MAF<0.005) variant. How would MAF filtering in the reference panels affect the attack? Could that be a possible protective measure? Have authors tried seeds with higher MAF thresholds for the low frequency variant? How important could this threshold be for successful reconstructions?

9. The RF classifier needs a bit more explanation and detail (P14:L30-46). How good is the performance of this classifier?

10. How is the inference of complete reconstruction done exactly in the "sieve" step (P14:L58-62)? Based on average dose?

11. How are the thresholds in the "extend" step defined? Authors need to be more specific rather than indicating close to 0.5, 0 or 1. In addition, how does this generalise to more matches? For example, for a query matching 4 reference haplotypes, do you expect 0.25 average dose?

12. The details of the discrete genotype prediction section can be explained a bit more clearly. For instance, how is the k chosen? Were different values (15-30) for different algorithms based on the ground truth accuracy?

13. Authors could provide a reference for the kinship coefficient.

14. It's quite an interesting application of the EM algorithm for the linking problem. As a minor comment/question, the link parameters theta is constrained to be binary in the probabilistic model but I wonder whether this is really necessary. For the M step, authors state that they round the solutions and the optimal solution always coincided with an integer. Does this mean values were always close to an integer? For better generalisation, why not do the rounding after all the iterations before the actual linking?

15. Fixed conditional distribution over the kinship coefficients for different degrees of relatedness was calculated empirically from the data. Authors correctly state that these values are in theory should be mainly deterministic but in practice, realised kinship varies a lot and IBD estimation for the kinship coefficient is an important factor. Outside the scope of this research and possibly inconsequential but the empirical distribution estimation from the same data might be causing some level of overfitting.

16. It is interesting to see that the initial input predictions for the EM algorithm were already good and only 1 iteration was needed for the chunk experiment. Have authors tried first initialising the EM algorithm with random parameters (instead of assuming no prior) or relatedness? Would it be difficult to converge to good local maximum likelihood in that case?

17. Authors could provide a graph representation for the minimum-cost flow algorithm.

18. Some references need to be fixed (e.g. 12, 20).

**Authors Response**

**Point-by-point responses to the reviewers’ comments:**

Reviewer #1:

It is a very interesting study to explore the risk of personal privacy leakage in the genotype imputation reference panel from an attacker's perspective. The authors focus on analyzing the latest version of the minimac imputation algorithm used by most imputation servers, including MIS and TIS, and investigate three other prominent imputation algorithms, including the PBWT algorithm used by the Sanger imputation service. The authors recommend how genotype imputation online services can mitigate the identified risks. Overall, the research is of great value in driving the balance between genomic data sharing and protecting privacy. The methods are appropriate to the study's aims, and the conclusions are adequately supported by the data shown. The work represents a significant advance and has broad interest to others in the field.

*Response: We thank the reviewer for kindly recognizing the significance and broad interest of our work.*

There are two comments:

1) While a series of recommendations are proposed to guide genotype imputation online services on how to mitigate privacy risks, there is a need to increase the discussion of whether suitable algorithms can ensure maximum use and avoid the possibility of privacy leakage.

*Response: We have expanded our Discussion and Conclusions to include a mention of differential privacy techniques, which provide a potential algorithmic solution to the privacy leakage demonstrated in our work. However, there remain key technical challenges to this approach, and at this time we are unfortunately not aware of any suitable algorithm within reach that can both achieve practical imputation accuracy and avoid privacy leakage. We believe some amount of leakage is inherent to the task of imputation regardless of the algorithm if we were to maintain accuracy of results. We hope our work motivates further research on effective privacy-protection strategies.*

2) In Figure 2, it can be seen that the results obtained from different reference panels are very different, and the authors need to analyze the internal reasons.

*Response: We have performed two new experiments to provide deeper insights into the differences among various reference panels. Firstly, we assessed our attack strategy against reference panels that represent the same underlying population but differ in size (1250, 2500, and 5012 haplotypes), derived by subsampling the 1KG dataset. Secondly, we compared panels of identical size (2500 haplotypes) but sourced from different ancestral populations (mixed population from 1KG, Black or African Americans, and Asian Americans). The results are provided in Figures S5, S6, and S20 and the accompanying analysis is provided in Supplementary Note 1. We have also updated the Results section to discuss these results.*

*A brief summary of findings from our new experiments:*

*Effect of panel size on reconstruction performance*

*● Smaller panels required fewer queries before redundant reconstruction caused the curve to level out (in Figure 2), resulting in a sharper curvature.*

*● The rate of correct reconstruction improved as panel size increased, likely because larger panels more frequently resulted in queries with unique matches.*

*● Similarly, the rate of incorrect reconstruction was found to be inversely related to panel size. Incorrect results primarily occurred due to queries being incorrectly predicted to have a single match, when they have no match but several close matches. These misclassifications occurred more frequently with smaller panels.*

*Effect of ancestral composition on reconstruction performance*

*● Queries with unique matches were constructed more frequently for panels with fewer (or more targeted) populations. This is likely due to the associated MAF data being more closely aligned with the reference panel, which increases the quality of the query seeds.*

*● However, these focused panels also had a higher rate of misclassification of queries that matched a small number of haplotypes, likely owing to the greater number of closely-matching queries for more homogeneous panels.*

*In conclusion, the reasons for differences in reconstruction performance across panels are complex, involving factors such as panel size, ancestry composition, and accuracy of MAF data. Our new results shed light on some of these factors, but the focus of our work remains to be demonstrating the feasibility of reconstruction in a diverse range of settings. We believe further improvements to the query selection strategy and classifier are possible, and such efforts are likely to enhance reconstruction rates beyond what we reported in our analyses.*

*As a side note, during the course of the above investigation, we discovered a simple modification of our query selection strategy (namely, disallowing queries that are too close to the chromosome boundaries) that consistently and significantly reduced the amount of incorrect reconstructions. We have updated our data, including Figure 2, with improved results reflecting this new strategy.*

Reviewer #2:

In this study, authors propose methodology to obtain reconstructed haplotypes from reference panels of public imputation servers via adversarial queries. In addition, they propose an algorithm to link the reconstructed haplotypes to form individual genomes. Imputation servers are commonly used for genomic imputation and allow researchers to impute their own genotype datasets without the need of direct access to high quality reference panels, hence detection of any possible privacy risk is of high importance for the development of adequate measures. Therefore, this work could be of broad interest to the genomics community. The manuscript is well written with extensive methodological explanation and mainly convincing (or with regard to the privacy context, concerning) results. Although I find this work interesting and valuable, there are some moderate/minor points which I believe need to be addressed.

*Response: We thank the reviewer for the thoughtful review. We have done our best to address all the points raised.*

1. The authors indicate that the imputation servers and relevant parties were informed (but in acknowledgements, they state that some servers will be notified before the publication). In what manner were they informed? Were they provided with possible protective measures indicated in the manuscript? Was there any response from these parties? Do authors think enough time was provided in case the relevant parties want to implement possible protective measures before this manuscript goes online? Although the imputation algorithms are publicly available, this work also involves server queries so I believe a green light from the relevant parties is important, especially considering the sensitivity of the subject.

*Response: In October 2022, we held a virtual meeting with relevant parties associated with the Michigan server and the TOPMed server, which are based on the same imputation software and analysis settings that we focused on in our work. We shared the key results and conclusions from our investigation as well as a draft of our manuscript. We discussed the mitigating measures as described in the manuscript.*

*The parties appreciated the advance notice and expressed the need to separately assess the risks in the context of their own reference panels, which we avoided in our work to minimize the impact on the existing servers while demonstrating the realistic nature of the threat. We are aware that there is ongoing discussion among the parties in this regard, and we agreed to provide assistance as needed. The parties have requested to be informed of a tentative publication date, but otherwise wished us good luck with the publication of this manuscript. In addition, we plan to inform the Sanger imputation service and ChinaMAP server of our findings upon acceptance of our manuscript, following any editorial guidance as needed. Although we did not directly study the settings of these additional servers, we believe it is appropriate to inform them in advance once we know our work will be published.*

2. It is stated that the minimac imputation algorithm gives extreme dosage values to the queried variants if they match exactly with one reference haplotype. What is the extent of "extreme" here? Authors show one example in Fig. S1 but it would be interesting to see the distribution of the assigned value and how much it varies with the increasing number of matches. Did authors check this or is there some reference to point to? Although the algorithm is explained in the methods, empirical or referential support for this statement could improve the manuscript since the motivation is built on it.

*Response: We have added a new figure (Figure S2) showing the empirical dosage distribution for queries with 1 through 5 matches or falling in the Other category (having more than 5 matches or no matches). These correspond to the categories used by our query classifier. Each distribution summarizes 100 examples belonging to the same category. Our new figure clarifies the extent of clear segregation of dosage values toward 0 and 1 for queries with unique matches. We have added a mention of this additional data in the Results section where we introduce haplotype reconstruction.*

3. Related to the previous point, it would be useful to get a statistical measure for the authors' observations (P4: L43-48). I believe this is not a single observation?

*Response: In a test of 100 randomly sampled queries on chromosome 20 each matching exactly one haplotype in 1000 Genomes Phase 3, we found that all 100 reconstructed the matching haplotype with no mistakes in one run of imputation. We have added a supplementary figure (Figure S3) visualizing these results and referred to it in the Results section, just after the sentences indicated by the reviewer.*

4. Authors claim 80% of 1KG haplotypes were outputted by their reconstruction algorithm but it's not clear whether these were exact matches (P4: L53). For all the reconstructed haplotypes, do authors mean complete reconstructions? If not, what is the percentage of these? Related to this point, "correct" match was defined as having less than 100 genotype differences from the reference haplotype but authors do not justify this arbitrary choice. Overall, performance metrics need further clarification.

*Response: The 80% refers to the percentage shown in Fig. 2A, representing complete chromosome-length reconstructions below the 100 mismatch threshold. As far as choosing 100, we chose a small, non-zero number so that nearly-perfect reconstructions would still be considered correct. We have added a supplementary figure (Figure S4) to demonstrate that the vast majority of reconstructed haplotypes are either perfect matches (i.e., zero mistakes) or have many mistakes (>1,000), so any cutoff in the range of 0 to 500 could have been chosen with little effect on the results as visualized in Fig. 2 and the exact choice is not particularly important. We have clarified these points near the indicated line and in the Fig. 2 caption.*

5. It is difficult to assess whether attack effectiveness changes with panel size with the available information (P5: L33-37). There are too many variables (like the variation between the reference panels) and figure 2 shows some difference in reconstructed haplotypes at 200K queries. In addition, there is the factor of stochasticity and the redundant outputs as the authors mention.

*Response: We thank the reviewer for this comment. We have conducted additional experiments to provide deeper insights into the various factors underlying reconstruction performance. Please see our response to Reviewer 1’s second comment for a detailed response.*

6. Both for the reconstruction and haplotype linking, is it possible for an adversary to obtain significance for the outputs? In other words, is there a way to assign reliability scores to the reconstructions without access to the reference panel? For haplotype linking, could the proposed population genetic model be used for this?

*Response: Yes, it would be possible for an adversary to obtain a measure of confidence associated with the output of our attack strategy. For reconstruction, a potential approach would be to feed back different portions of an output back into imputation. If the output really does match a haplotype in the reference panel, the new output should be nearly identical to the original. For linking, the probabilistic nature of our algorithm (which incorporates the population genetic model) allows one to interpret the likelihood score associated with each haplotype set as a measure of linking quality. We have added a mention of these extension ideas in Methods.*

7. A brief discussion/information on time and computational requirements could be useful since they would be important factors for an adversarial attack.

*Response: We conducted additional experiments to better understand running time and computational requirements. Results concerning haplotype reconstruction are shown in Figure S15. Those concerning haplotype linking are shown in Figure S16. The running time of haplotype reconstruction scales linearly with the number of queries imputed, and the greater part of the running time is time for imputation, which depends on the resources and latency of the imputation server, rather than on the adversary’s resources. The running time and memory usage for the haplotype linking algorithm scales linearly with the number of target set haplotypes and with the number of relative set samples. We have added a paragraph on these considerations to the Discussion and Conclusions section of the manuscript.*

8. Imputing rare alleles is notoriously difficult and authors' attack queries are built around a very low frequency (MAF<0.005) variant. How would MAF filtering in the reference panels affect the attack? Could that be a possible protective measure? Have authors tried seeds with higher MAF thresholds for the low frequency variant? How important could this threshold be for successful reconstructions?

*Response: We conducted two additional experiments, in which we filtered out rare variants in the reference panel with MAFs below 0.5% or 1%. The results are summarized in Figure S17. In both cases, we configured the attack to use remaining variants with MAF<2.5% to include in the query seeds. We found that constraining the attack to use higher MAF variants in the query does slow down reconstruction, but only to a limited extent: for example, around 20% of the reference panel is reconstructed for both 0.5% and 1% MAF thresholds, in contrast to 30% without filtering using the same number of queries. Moreover, introducing such a filtering would come at a high cost of utility, since it would prevent a large number of relatively rare variants (e.g., with MAF<1%) from being imputed, thus precluding them from the downstream analyses. We discuss these considerations in the “Haplotype reconstruction experiments” section of Methods, in the “Attack settings” subsection. We also indicate in our Discussion that MAF filtering could be used as a mitigation strategy to slow down the attack.*

9. The RF classifier needs a bit more explanation and detail (P14:L30-46). How good is the performance of this classifier?

*Response: We have added additional details to our explanation of the classifier in the Methods subsection “Step 2: Sieve,” including a more exact specification of the dosage buckets it uses to construct the input feature vectors. We have also added a supplementary figure showing the performance of the classifier on a test dataset, as Figure S18, and we provide classifier performance evaluation in the context of full reconstruction experiments in Figures S5 and fS6. We would like to emphasize that the classifier used in our experiments serves only as an example of what may be possible (and one that leads to sufficiently accurate reconstruction performance). More accurate classifiers could likely be developed to further improve the attack strategy.*

10. How is the inference of complete reconstruction done exactly in the "sieve" step (P14:L58-62)? Based on average dose?

*Response: Complete reconstruction is determined by the classifier. The classifier takes a feature vector summarizing the imputed dosages of each query as input and predicts if the query has 1 through 5 matches or falls in the Other category (having more than 5 matches or no matches). If the query is predicted to have a single match, then it is considered a complete reconstruction and thus output by the algorithm. We have updated the text in the indicated section to clarify these points.*

11. How are the thresholds in the "extend" step defined? Authors need to be more specific rather than indicating close to 0.5, 0 or 1. In addition, how does this generalise to more matches? For example, for a query matching 4 reference haplotypes, do you expect 0.25 average dose?

*Response: The output of a query matching n haplotypes is characterized by concentrations of dosages at multiples of 1/n, which correspond to the proportions of REF vs. ALT alleles among the matches (e.g., with 4 matches and only 1 with the ALT allele, we expect a dosage near 0.25). We described dosage values as being “close” to certain thresholds only to convey the intuition behind our strategy. The actual classification of dosage outputs into query categories with different numbers of matches is performed by the random forest classifier. We have further clarified the details of the classifier in Methods.*

12. The details of the discrete genotype prediction section can be explained a bit more clearly. For instance, how is the k chosen? Were different values (15-30) for different algorithms based on the ground truth accuracy?

*Response: A higher k generally results in fewer reconstruction mistakes, at the cost of requiring a greater number of runs of imputation, so its exact setting is simply a trade-off between the number of imputation runs and the rate of successful reconstruction. The key finding from our analysis in the indicated section — that outputting only the discrete genotypes does not prevent reconstruction attempts — does not depend on the choice of k. The example choices in our experiments (15-30) account for different baseline reconstruction rates among different imputation algorithms as observed in small test experiments, where we attempted to uniformize the settings across the methods. An adversary could use the same value of k we used for each method or attempt to further tune the trade-off based on a public reference panel (e.g. 1KG). We have updated the “Alternative strategy for discrete genotype predictions” subsection in Methods to discuss these points.*

13. Authors could provide a reference for the kinship coefficient.

*Response: We have added a reference for the kinship coefficient in the Methods subsection “Capturing haplotype-diplotype relatedness via semi-kinship (SK) coefficients”.*

14. It's quite an interesting application of the EM algorithm for the linking problem. As a minor comment/question, the link parameters theta is constrained to be binary in the probabilistic model but I wonder whether this is really necessary. For the M step, authors state that they round the solutions and the optimal solution always coincided with an integer. Does this mean values were always close to an integer? For better generalisation, why not do the rounding after all the iterations before the actual linking?

*Response: The original formulation of the problem considers binary theta since the true linkage patterns are inherently discrete. However, we solve a relaxation of this problem with continuous theta between 0 and 1 for tractability, since working with integer variables necessitates combinatorial optimization which is generally challenging to perform. We do not round the theta in each M step and only round after all iterations, as the reviewer suggested. We have made edits to clarify these points in the Methods subsection “Minimum-cost network flow algorithm for linkage optimization (M-step).” As we also noted in that subsection, in our experiments we found this rounding step not to result in approximation, as solutions already consisted of integer values.*

15. Fixed conditional distribution over the kinship coefficients for different degrees of relatedness was calculated empirically from the data. Authors correctly state that these values are in theory should be mainly deterministic but in practice, realised kinship varies a lot and IBD estimation for the kinship coefficient is an important factor. Outside the scope of this research and possibly inconsequential but the empirical distribution estimation from the same data might be causing some level of overfitting.

*Response: In order to establish that the algorithm could successfully leverage conditional semi-kinship coefficient distributions from an external dataset, we conducted an additional experiment in which we split our dataset sample-wise into two equal-sized, non-overlapping groups, estimating distributions based on one group and running our haplotype linking algorithm on the other. We found that the performance under this new setting remained close to the original experiment, indicating that our results do not rely on data-specific kinship coefficient distributions. We have included the results of this experiment in Figure S19 and referred to it in the haplotype linking algorithm Methods section, in the “Implementation details” subsection.*

16. It is interesting to see that the initial input predictions for the EM algorithm were already good and only 1 iteration was needed for the chunk experiment. Have authors tried first initialising the EM algorithm with random parameters (instead of assuming no prior) or relatedness? Would it be difficult to converge to good local maximum likelihood in that case?

*Response: As the reviewer alluded to, local optimization algorithms such as the EM algorithm depend on a good initialization due to the existence of local optima. We have not tried random initialization given easy access to heuristic approaches for constructing a reasonable starting point. It is indeed interesting that 1 iteration was sufficient in our test case, but the quality of the solution substantially improved after this iteration, as can be seen by comparing Figure S11B (initialization) with Figure 3B (after the EM algorithm).*

17. Authors could provide a graph representation for the minimum-cost flow algorithm.

*Response: We have added a graphical representation as Figure S9 and referred to it both in the main text and in the “Probabilistic inference algorithm for haplotype linking” subsection of Methods.*

18. Some references need to be fixed (e.g. 12, 20).

*Response: We have fixed references that were improperly formatted.*

**Second round of review**

**Reviewer 1**

The provided text discusses a potential privacy risk related to genetic data imputation services. The authors describe an attack strategy involving haplotype reconstruction and haplotype linking, which could allow an adversary to reconstruct genetic information from reference panels and potentially link this information to specific individuals, even in cases where data use agreements and access controls are in place. The attack strategy is outlined, and experimental results are presented to demonstrate its effectiveness. The authors emphasize the need for awareness and mitigation of this privacy risk in the genomics community, which is very insightful and helpful. Several suggestions:

1. If consider implement variant filtering measures to exclude rare or sensitive variants from reference panels used for imputation, which could limit the potential for attackers to reconstruct specific genomic information.

2. If consider behavioral analysis, employing algorithms to monitor user behavior and identify suspicious patterns of interaction with imputation services. Unusual or anomalous behavior could trigger alerts for further investigation. Such as rate-limiting mechanisms to prevent bulk queries or repeated requests from a single user. This could slow down potential attackers and reduce the risk of data reconstruction.

3. In the method section step two, why random forest is chosen to classify? Is there any comparation to other classifiers?

4. It is important to encourage collaborative research among the genomics community to address privacy risks. This includes sharing findings, best practices, and potential countermeasures to collectively protect genetic data. What is the ideal balance between providing valuable genetic data services and safeguarding individual privacy and how to get it in the future may be needed to be discussed.

**Reviewer 2**

I thank the authors for their effort, I believe they responded satisfactorily to all the comments with multiple additional analyses and clarified all the points raised. I especially appreciate the communication with the relevant parties given the sensitivity of this work, analyses on the performance of the attack for different reference panel characteristics and clarifications in performance metrics. I believe the manuscript improved substantially with the new analyses and edits, and I'm satisfied with the current state of the manuscript. Below are a few typographical errors I was able to notice.

- P17 - "...original, in one setting it to the ALT allele..."

- Figure S17 - "30 OF 1KG%"

- Figure S20 caption - "...shows an a query..."

**Authors Response**

**Point-by-point responses to the reviewers’ comments:**

Reviewer #1:

The provided text discusses a potential privacy risk related to genetic data imputation services. The authors describe an attack strategy involving haplotype reconstruction and haplotype linking, which could allow an adversary to reconstruct genetic information from reference panels and potentially link this information to specific individuals, even in cases where data use agreements and access controls are in place. The attack strategy is outlined, and experimental results are presented to demonstrate its effectiveness. The authors emphasize the need for awareness and mitigation of this privacy risk in the genomics community, which is very insightful and helpful. Our previous comments have been addressed in the revised version. Overall, we have no more comments except several suggestions for the revised manuscript.

Several suggestions:

1. If consider implement variant filtering measures to exclude rare or sensitive variants from reference panels used for imputation, which could limit the potential for attackers to reconstruct specific genomic information.

*Response: We appreciate the suggestion. We note in our Discussion section how filtering rare variants by MAF from panels could slow the attack somewhat at the cost of being able to impute those variants, with the results of our tests of this strategy displayed in Figure S17. It is a good point that sensitive variants could also be excluded from reference panels, and we have added a comment about this to our Discussion.*

2. If consider behavioral analysis, employing algorithms to monitor user behavior and identify suspicious patterns of interaction with imputation services. Unusual or anomalous behavior could trigger alerts for further investigation. Such as rate-limiting mechanisms to prevent bulk queries or repeated requests from a single user. This could slow down potential attackers and reduce the risk of data reconstruction.

*Response: We note in our Discussion section that an imputation server could consider a user’s full course of interaction with the server and thereby potentially spot suspicious behavior suggestive of a reconstruction attack. We also mention in the Discussion section that rate-limiting queries could make the attack less practical due to the large number of queries it requires, though we note that an adversary could circumvent this to an extent by using multiple accounts or colluding with other users.*

3. In the method section step two, why random forest is chosen to classify? Is there any comparation to other classifiers?

*Response: We proceeded with a random forest classifier with the particular settings we did because it yielded successful results. We did not compare extensively with other types of classifiers because optimization was not our primary aim, and it is certainly possible that performance could be improved somewhat via a different type of classifier or one with adjusted settings.*

*However, we expect that such improvements would be limited, since the performance is already quite good and the types of misclassifications we discuss in Supplementary Note 1 would likely trouble the classifier regardless of type and settings. We have added an additional brief note about our choice in the “Step 2: Sieve” subsection of the “Seed-Sieve-Extend (SSE) strategy for haplotype reconstruction” section in Methods.*

4. It is important to encourage collaborative research among the genomics community to address privacy risks. This includes sharing findings, best practices, and potential countermeasures to collectively protect genetic data. What is the ideal balance between providing valuable genetic data services and safeguarding individual privacy and how to get it in the future may be needed to be discussed.

*Response: We feel that we have thoughtfully described these issues in the Discussion section concerning the imputation servers. We believe the mitigation strategies we suggest could shift the situation for the better in the direction of increased privacy, but as we note at the end of the Discussion, any mitigations are likely to come at the cost of some utility and we do not believe that with existing strategies the risk can be entirely eliminated. In our Conclusion, we encourage further research aimed at developing effective mitigation strategies and assessing our current and future data sharing mechanisms.*

Reviewer #2:

I thank the authors for their effort, I believe they responded satisfactorily to all the comments with multiple additional analyses and clarified all the points raised. I especially appreciate the communication with the relevant parties given the sensitivity of this work, analyses on the performance of the attack for different reference panel characteristics and clarifications in performance metrics. I believe the manuscript improved substantially with the new analyses and edits, and I'm satisfied with the current state of the manuscript. Below are a few typographical errors I was able to notice.

- P17 - "...original, in one setting it to the ALT allele..."

- Figure S17 - "30 OF 1KG%"

- Figure S20 caption - "...shows an a query..."

*Response: We appreciate the reviewer’s attention to detail in spotting these mistakes. We have corrected all three.*
